# Constructing Stable Bifunctional Electrocatalyst of Co─Co_2_Nb_5_O_14_ with Reversible Interface Reconstitution Ability for Sustainable Zn‐Air Batteries

**DOI:** 10.1002/advs.202413796

**Published:** 2025-03-11

**Authors:** Shuo Chen, Liang Zhang, Zheng Liu, Yuehui Chen, Shouzhu Li, Yuanyuan Zhang, Junyu Chen, Jianhua Yan

**Affiliations:** ^1^ College of Textiles Donghua University Shanghai 201620 China; ^2^ Xinjiang Key Laboratory of New Energy and Energy Storage Technology Xinjiang Institute of Technology Akesu 843100 China; ^3^ College of Textiles & Clothing Qingdao University Qingdao 266071 China; ^4^ College of Textiles & Garments Hebei University of Science and Technology Shijiazhuang 050018 China

**Keywords:** bifunctional ORR/OER electrocatalyst, Co nanodots, Co_2_Nb_5_O_14_ nanorods, dynamically reversible catalytic Interface, rechargeable Zn‐air batteries

## Abstract

Transition metal and metal oxide heterojunctions have been widely studied as bifunctional oxygen reduction/evolution reaction (ORR/OER) electrocatalysts for Zn‐air batteries, but the dynamic changes of transition metal oxides and the interface during catalysis are still unclear. Here, bifunctional electrocatalyst of Co─Co_2_Nb_5_O_14_ is reported, containing lattice interlocked Co nanodots and Co_2_Nb_5_O_14_ nanorods, which construct a strong metal‐support interaction (SMSI) interface. Unlike the recognition that transition metals mainly serve as ORR active sites and metal oxides as OER active sites, it is found that both ORR/OER sites originate from Co_2_Nb_5_O_14_, while Co acts as an electronic regulatory unit. The SMSI interface promotes dynamic electron transfer between Co/Co_2_Nb_5_O_14_, and the reversible active sites of Nb^4+^/Nb^5+^ realize bidirectional adsorption/migration of intermediates, thereby achieving dynamic reversible interface reconstitution. The electrocatalyst shows a high ORR half‐wave potential of 0.84 V, a low OER overpotential of 296.3 mV, and great cycling stability over 30000 s. The ZAB shows a high capacity of 850.6 mA h·gZn^−1^ and can stably run 2050 cycles at 10 mA·cm⁻^2^. Moreover, the constructed solid‐state ZAB also shows leading cycling stability in comparison with the previous studies.

## Graphical abstract

A bifunctional electrocatalyst of Co─Co_2_Nb_5_O_14_ with a strong interaction interface is fabricated, and a bidirectional adsorption/migration mechanism of oxygen species between the reversible active sites of Nb^5+^/Nb^4+^ has been proposed. With the catalytic strategy design, the liquid Zn‐air batteries exhibits long‐term stability over 2050 cycles, and the solid‐state Zn‐air batteries also exhibits leading long‐cycling stability.

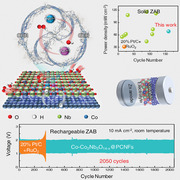

## 1 Introduction

The excessive consumption of fossil fuels has led to considerable societal challenges. The consequent shortage of resources and environmental degradation highlights the importance of advancing new technologies for energy storage and conversion.^[^ ^1^, ^2^ ^]^ Due to high energy density, environmental friendliness, and pollution‐free characteristics, rechargeable Zinc‐air batteries (ZABs) are regarded as a promising next‐generation battery system. ZABs are typically composed of metallic Zn as the anode, oxygen from the air as the cathode, and an alkaline electrolyte.^[^ ^3^, ^4^ ^]^ The ORR occurs during the discharge the OER takes place during charging. Both reactions involve a multi‐electron transfer process with complex mechanisms, including mass transfer across multiple phases and interfaces. These factors are key challenges that lead to poor kinetic performance and hinder the overall power and stability of ZABs.^[^ ^5^, ^6^ ^]^ It is thus imperative to develop efficient bifunctional electrocatalysts with high stability to advance the commercial development of ZABs.

Currently, Pt‐based precious metals and IrO_2_/RuO_2_‐based precious oxides are broadly used as efficient commercial ORR and OER catalysts, respectively, but the limited sources and poor stability of these catalysts hinder their sustainable development.^[^ ^7^ ^]^ Transition metals (TMs) and transition metal oxides (TMOs) have garnered extensive attention due to their abundant reserves and tunable electronic structures. However, the inherently high reaction energy barrier of TMOs and the easy degradation of TMs always lead to unsatisfactory bifunctional catalysis. ^[^ ^8^ ^]^ Researchers tried to construct TMs/TMOs interfacial structures to enhance the stability and lower the catalytic energy barrier of ORR/OER. For example, Kou reviewed the important role of charge transfer at the TMs/TMOs interface in previous studies.^[^ ^9^ ^]^ Nevertheless, the interfacial structure change and interaction of TMs/TMOs during the reversible ORR/OER processes are unclear. In particular, even the reducible metal ion pairs like Ti^4+^/Ti^3+^, Nb^5+^/Nb^4+^, and Mo^6+^/Mo^5+^, known as the most effective TMO catalysts, their dynamic changes during catalysis have often been overlooked.^[^ ^10^ ^]^ Therefore, it is crucial to precisely uncover the interactions between TMs and TMOs and thoroughly consider the dynamic structural reconstitution of reducible TMOs.

Herein, to understand the interfacial structure reconstitution and the interactions between TMs/TMOs, we design a stable and efficient bifunctional electrocatalyst that includes lattice interlocked Co nanodots (NDs) and Co_2_Nb_5_O_14_ nanorods (NRs) in porous carbon nanofibers (PCNFs), denoted as Co─Co_2_Nb_5_O_14‐x_@PCNFs. With simulation calculations and experimental verification, we found the stable and efficient bifunctional catalysis was from its dynamic reversible catalytic interface. For one thing, the unique lattice interlocked Co and Co_2_Nb_5_O_14_ established a strong metal‐support interaction (SMSI) interface, which promoted dynamic electron transfer between Co and Co_2_Nb_5_O_14_ and redistributed the local charge density to satisfy the different needs of ORR and OER. For another, the bidirectional adsorption and migration of oxygen intermediates on the active sites of Nb^5+^ and Nb^4+^ facilitated reversible ORR and OER catalysis. Moreover, the oxygen‐rich PCNF framework effectively protected the active sites and promoted mass transfer during reactions, thereby enhancing the catalytic efficiency and stability. As a result, the catalyst showed a high ORR activity with a half‐wave potential of 0.84 V and a low OER overpotential of 296.3 mV at 10 mA·cm^−2^, and much higher stability for both ORR and OER (the normalized current density decreased by only 6.55% and 6.97%, respectively after 30 000 s) than the commercial catalysts of 20% Pt/C and RuO_2_. Moreover, the as‐assembled liquid ZABs exhibited a high specific capacity of 850.6 mA h·g_Zn_^−1^ and could run 2050 stable cycles at 10 mA·cm⁻^2^, and the solid ZABs exhibited great flexibility and were much more stable than the previous reports.

## 2 Results

### 2.1 Catalytic Mechanism of Co─Co_2_Nb_5_O_14‐x_ and its Electrochemical Activity in ZABs

**Figure 1:**
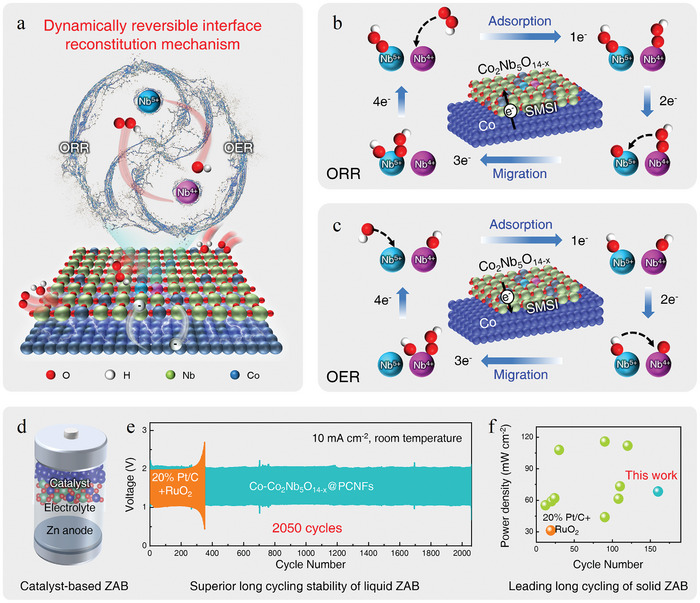
Catalytic mechanism and electrochemical activity of ZABs. a) Conceptual diagram of the catalytic mechanism. The proposed 4e^−^ catalytic mechanisms for b) ORR and c) OER. d) The structure of the prepared ZABs is based on the new catalyst. e) Superior long cycling stability of the as‐assembled liquid ZABs. f) Leading long cycling stability of the as‐assembled solid ZABs.

The lattice interlocked Co NDs and Co_2_Nb_5_O_14_ NRs endow the catalyst with a unique SMSI interface and a dynamically reversible interface reconstitution ability, as shown in **Figure** **1**a. The dynamic reversibility includes the dynamic bidirectional electron transfer through the SMSI interface and bidirectional adsorption/migration of intermediate oxygen species by the active site pairs of Nb^5+^/Nb^4+^, both of which enhance the catalytic stability and efficiency during the opposite ORR and OER process. The interpretation of the catalytic mechanism is shown in Figure 1b,c. In ORR, Nb^5+^ and Nb^4+^ act as the main active sites and adsorption sites, respectively. Nb^4+^ first adsorbs the oxygen intermediates of ·OOH at step 1 and then transfers it to the Nb^5+^ site upon completion of step 3, thereby facilitating the continuous adsorption and desorption of oxygen species at the reaction sites. Simultaneously, due to the presence of the SMSI interface, Co continuously supplies electrons to the reaction sites, promoting the transformation of oxygen species. The situation is the opposite for OER, where the oxygen intermediates of ·OH are first adsorbed by Nb^5+^ sites at step 1 and then transferred to the active sites of Nb^4+^ when step 3 is completed. Similarly, surplus electrons are transferred from the reaction sites to the interface to ensure the reaction proceeds smoothly.

Based on the bifunctional electrocatalyst of Co─Co_2_Nb_5_O_14‐x_@PCNFs, both liquid and solid‐type rechargeable ZABs were fabricated, and the schematic structure is shown in Figure 1d. The liquid ZABs exhibited superior longer‐term stability over 2050 cycles in comparison with the commercial catalyst of Pt/C‐RuO_2_ (Figure 1e). After 2050 cycles, the round‐trip efficiency was still as high as 52.7%. Figure 1f compares the performance of solid ZABs with other catalysts reported in recent years. The solid ZABs based on Co─Co_2_Nb_5_O_14‐x_@PCNFs exhibited leading long cycling stability and surpassed most Co‐based catalysts tested under similar conditions, confirming the superior advantages of the as‐designed catalyst.^[^ ^1^, ^4^, ^11^, ^12^, ^13^, ^14^, ^15^, ^16^, ^17^ ^]^

### 2.2 Materials Synthesis and Characterization

**Figure 2:**
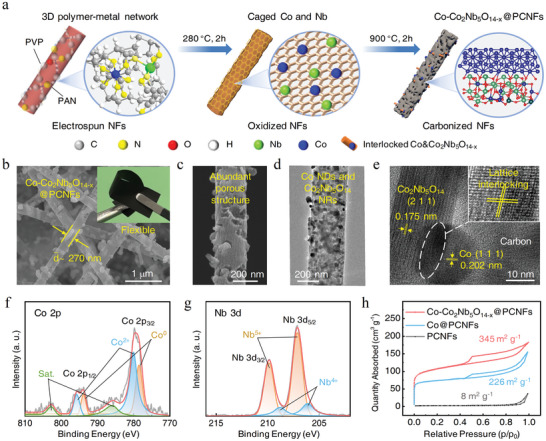
Materials synthesis and characterization. a) Material synthesis strategy. b,c) SEM images, and d,e) TEM images of the as‐fabricated Co─Co_2_Nb_5_O_14‐x_@PCNFs. XPS spectra of f) Co 2p and g) Nb 3d in Co─Co_2_Nb_5_O_14‐x_@PCNFs. h) N_2_ adsorption–desorption isotherms of the three materials.

**Figure** **2**a shows the process of preparing flexible Co─Co_2_Nb_5_O_14‐x_@PCNF films with sol‐gel electrospinning followed by pre‐oxidation and carbonization in the H_2_/Ar atmosphere. A stable polymer‐metal sol with a typical Tyndall effect was first prepared by dissolving the precursors of polyvinylpyrrolidone (PVP), polyacrylonitrile (PAN), ethoxyniobium and acetylacetone cobalt in N, N‐Dimethylformamide (Figure S1a, Supporting Information). Fourier transform infrared (FTIR) spectrometer was applied to analyze the chemical interactions in the sol. Compared with the pure PAN and PVP solutions, there were no new chemical bonds for the PAN/PVP mixture (Figure S1, Supporting Information), but the addition of acetylacetone cobalt introduced new peaks at 1584, 1518, 1015, and 925 cm^−1^, and the addition of ethoxyniobium also introduced a new peak at 1518 cm^−1^ (Figure S1c, Supporting Information). Since the peak at 1518 cm^−1^ was attributed to the telescopic vibration of ─C═N and considering the high polarity and coordination ability of the ─C≡N (2245 cm^−1^) group in PAN, it was believed that PAN complexed with Co^2+^ and Nb^5+^ in 6‐ and 5 fold coordination states, respectively.^[^ ^18^, ^19^, ^20^ ^]^ This was supported by the solvatochromism in Figure S1a (Supporting Information), the ─C≡N formed a 6‐ligand complex with Co^2+^, resulting in a purple hue in the solution. Therefore, in the polymer‐metal sol system, PAN also served as a complexing agent with the metal ions and caging them within its network.

Subsequently, the precursor sol was electrospun into NF films (Figure S2a, Supporting Information) followed by pre‐oxidation in air (Figure S2b, Supporting Information). After pre‐oxidation, the peaks at 1674 and 1292 cm^−1^ corresponding to PVP, and at 1015 and 925 cm^−1^ corresponding to acetylacetone groups disappeared (Figure S2c, Supporting Information), suggesting that PVP and PAN were partially decomposed.^[^ ^21^ ^]^ Moreover, the characteristic peaks of ─C≡N and ─C─N at 2245 and 1518 cm^−1^ also disappeared, indicating that ─C≡N and ─C─N could interact with metal cations to promote the cyclization reaction of PAN and thus further caged metal cations, which prevented overgrowth of Co NDs and Co_2_Nb_5_O_14_ NRs, and thus stabilized the catalyst structure during carbonization (Figure S2d, Supporting Information).^[^ ^22^, ^23^ ^]^ The carbonization process was explored by thermogravimetric (TG) analysis (Figure S2e, Supporting Information). Initially, a mass decline occurred from 25 to 238 °C due to the evaporation of solvents, and PVP decomposed from 238 to 362 °C. The PVP content was positively correlated to the number of pores on the fiber surface, but the NF structure would collapse when its content exceeded a critical value (Figure S3, Supporting Information). Then, from 352 to 560 °C, PAN formed a carbon framework by carbon thermal reduction, which played an important role in supporting the monolithic structure of NFs. Finally, from 560 to 990 °C, both H_2_, and carbon reduced Co^2+^ to Co^0^, accompanied by the formation of defective Nb_2_O_5‐x_. The high‐valence Nb^5+^ could anchor Co^0^, where the Co atom occupied the Nb^5+^ position, and thus created numerous surface vacancies (O_vac_) and Nb^4+^.^[^ ^24^ ^]^ This was confirmed by X‐ray diffraction (XRD) with varying Co/Nb ratios, as shown in Figure S4 (Supporting Information). Due to the confinement effect of PAN and the high‐valence Nb, adjacent Co NDs and Co_2_Nb_5_O_14_ NRs underwent grain boundary fusion to form stable lattice interlocked SMSI interfaces. The SEM images and XRD patterns of materials subjected to carbonization at different temperatures are shown in Figure S5 (Supporting Information). The NFs contained T‐Nb_2_O_5_, M‐Nb_2_O_5_, and Co at 800 °C. The Co_2_Nb_5_O_14_ phase appeared at 900 °C, but its peak intensity decreased greatly at 1000 °C since it was reduced to NbO_2_.^[^ ^25^ ^]^

Figure 2b,c and Figure S6 (Supporting Information) show the scanning electron microscopy (SEM) images of Co─Co_2_Nb_5_O_14‐x_@PCNFs, PCNFs, Co@PCNFs, NbO_2_@PCNFs, and Nb_2_O_5_@PCNFs, where the latter four were studied as controls. The Co─Co_2_Nb_5_O_14‐x_@PCNFs with a porous structure contained many embedded NDs and NRs, and the average NF diameter was ≈270 nm. The transmission electron microscopy (TEM, Figure 2d,e; Figure S7, Supporting Information) images reveal that Co NDs and Co_2_Nb_5_O_14_ NRs were evenly dispersed and formed a distinct lattice interlocked SMSI structure. It was reported that the SMSI could stabilize metal and modulate interfacial reactions in catalysis.^[^ ^26^ ^]^ The average size of Co NDs was 22.86 nm, which was much smaller than that of Co@PCNFs, highlighting the size limitation effect of SMSI. From the energy dispersive spectroscopy (EDS, Figure S8, Supporting Information), metal elements were evenly distributed in NFs and the atomic ratio of Co/Nb was close to 1:1. The selected area electron diffraction (SAED) pattern confirmed that Co─Co_2_Nb_5_O_14‐x_@PCNFs had a polycrystalline structure (Figure S9, Supporting Information).

The structure and phase changes of catalysts were then analyzed using XRD and X‐ray photoelectron spectroscopy (XPS). The XRD refinement (Figure S10, Supporting Information) confirmed that the Co─Co_2_Nb_5_O_14‐x_@PCNFs were primarily composed of Co (55.79%, PDF#15‐0806) and Co_2_Nb_5_O_14_ (29.64%, PDF#42‐0422), with a small amount of Nb_2_O_5_ (14.57%, PDF# 30–0873). Figure S11 (Supporting Information) shows the XRD results of the four control catalysts. The XPS spectra for Co 2p confirmed the existence of Co^0^ and Co^2+^ (Figure 2f), and the content of Co^2+^ was much higher than that of Co@PCNFs (Figure S12a, Supporting Information). The spectra of Nb 3d for Co_2_Nb_5_O_14‐x_@PCNFs (Figure 2g) displayed the coexistence of Nb^5+^ and Nb^4+^, and the XPS spectra of Nb_2_O_5_@PCNFs and NbO_2_@PCNFs confirmed the composite valence states of Nb (Figure S13, Supporting Information). From the spectra of O 1s (Figure S12b, Supporting Information), the catalyst also contained abundant O_vac_. Additionally, the content of C─O─C in Co─Co_2_Nb_5_O_14‐x_@PCNFs was much higher than that in Co@PCNFs by comparing the C 1s spectra, confirming the oxygen‐enriched carbon framework in the catalyst (Figure S12e,f, Supporting Information), which could promote the OER catalysis.^[^ ^27^ ^]^ The XRD and XPS results proved that during the carbonization Co^0^ replaced Nb^5+^ in Nb_2_O_5_ to form Co_2_Nb_5_O_14‐x_ with abundant Nb^4+^ and O_vac_. From thermogravimetric (TG, Figure S14a, Supporting Information) and inductively coupled plasma (ICP, Figure S14b, Supporting Information), the loading of Co NDs in Co─Co_2_Nb_5_O_14‐x_@PCNFs reached 11.98 wt.%, and the carbon content was ≈39.10 wt.%.

The specific surface area (SSA) and pore types of the catalysts were determined by N_2_ adsorption isotherms (Figure 2h). All samples displayed typical Type IV isotherms, classifying them as mesoporous adsorbent materials.^[^ ^28^ ^]^ This was further corroborated by the pore size distribution (Figure S15, Supporting Information). The SSAs of Co─Co_2_Nb_5_O_14‐x_@PCNFs, Co@PCNFs, and PCNFs were calculated as 345, 226, and 8 m^2^·g^−1^, respectively. The dispersed Co NDs and Co_2_Nb_5_O_14_ NRs, coupled with the oxygen‐rich defective carbon framework contributed to the large SSA and pore volume of Co─Co_2_Nb_5_O_14‐x_@PCNFs. The large SSA contributed to an increase in the exposed active sites, and the abundant mesoporous structure could promote the transport of electrolytes, O‐species, and electrons.^[^ ^29^ ^]^ The Raman spectra of all three samples (Figure S16, Supporting Information) exhibited two characteristic peaks at 1338 cm^−1^ (D‐band) and 1580 cm^−1^ (G‐band). The D‐band associated with defects and disorders in carbonaceous materials could enhance the OER activity, and the G‐band associated with in‐plane stretching vibration of sp^2^ hybridized carbon could improve the ORR activity.^[^ ^30^, ^31^ ^]^ The Co─Co_2_Nb_5_O_14‐x_@PCNFs exhibited the highest I_D_/I_G_ value of ∼0.9908 due to its oxygen‐enriched defective carbon.

### 2.3 Electrocatalytic ORR/OER Performance

**Figure 3:**
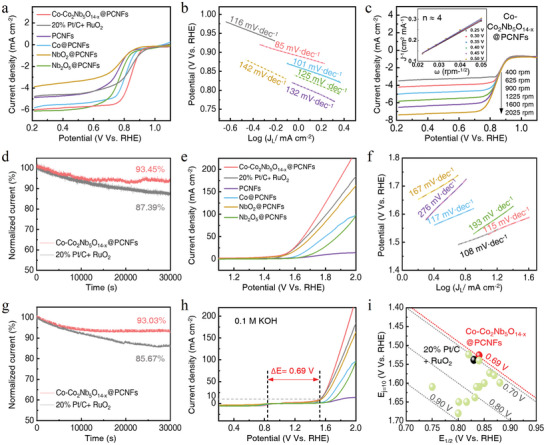
Electrocatalytic ORR/OER performance. a) ORR LSV curves that show the E_1/2_ of the six catalysts at a rotation speed of 1600 rpm. b) Tafel plots of the six electrodes at 1600 rpm. c) LSV curves at different rotation speeds of Co─Co_2_Nb_5_O_14‐x_@PCNFs. The inset figure was the K−L plot at different potentials. d) ORR stability testing of the catalysts by chronoamperometry at 0.6 V Vs. RHE. e) OER LSV curves of the six catalysts. f) Tafel plots of the six electrodes. g) OER stability testing of the catalysts at 1.6 V Vs. RHE. h) The bifunctional indicator of ORR and OER. i) Comparison of ΔE values between Co─Co_2_Nb_5_O_14‐x_@PCNFs and other catalysts.

The ORR and OER catalysis were evaluated with a three‐electrode test system in an O_2_‐saturated alkalic solution (0.1 m KOH). The catalysts were evenly coated on a glassy carbon electrode for testing ORR (Figure S17, Supporting Information). The cyclic voltammetry (CV) curves performed at a scan rate of 50 mV·s^−1^ showed that all samples displayed obvious reduction peaks in the O_2_‐saturated solution, confirming their effective ORR activities (Figure S18, Supporting Information). The linear scanning voltammetry (LSV) curves were measured at 1600 rpm to disclose the ORR kinetics. As shown in **Figures** **3**a and S19a (Supporting Information), the Co─Co_2_Nb_5_O_14‐x_@PCNFs exhibited the highest ORR activity among the samples, with a half‐wave potential (E_1/2_) of 0.84 V, which was better than that of Co@PCNFs (0.81 V), NbO_2_@PCNFs (0.75 V) and Nb_2_O_5_@PCNFs (0.77 V). The LSV curves of the commercial catalyst (0.83 V) and PCNFs (0.76 V) without deducting the background currents were also plotted for comparison (Figure S20, Supporting Information). From the perspective of diffusion‐limited current density (DLCD, Figure S19b, Supporting Information), Nb_2_O_5_@PCNFs had the highest ultimate current density of 6 mA·cm^−2^, and NbO_2_@PCNFs showed the lowest value of 3.69 mA·cm^−2^. The Nb^5+^ contributed to high DLCD in Co─Co_2_Nb_5_O_14‐x_@PCNFs. The corresponding Tafel plots of Co─Co_2_Nb_5_O_14‐x_@ PCNFs was 85 mV·dec^−1^ (Figure 3b), which was lower than those of the commercial catalyst (116 mV·dec^−1^), PCNFs (132 mV·dec^−1^), Co@PCNFs (101 mV·dec^−1^), NbO_2_@PCNFs (142 mV·dec^−1^), and Nb_2_O_5_@PCNFs (125 mV·dec^−1^), showing the fastest ORR kinetics.

To further explore the reaction pathways and mechanisms of ORR, the LSV curves were plotted at speeds from 400 to 2025 rpm (Figure 3c; Figure S21, Supporting Information), and the Koutecky–Levich (K–L) plots (inner figures) based on the relationship of J^−1^ and ω^−1/2^ were also plotted. The electron transfer number (n) for these catalysts was calculated as close to 4, except for Nb_2_O_5_@PCNFs (n ≈ 3). This finding was further confirmed by the RRDE tests (Figure S22, Supporting Information). A high n‐value and a low H_2_O_2_ yield were indicative of high 4e^−^ catalytic efficiency. The results at 0.6 V showed that Nb_2_O_5_@PCNFs had the lowest 4e^−^ catalytic efficiency with n = 2.86 and an H_2_O_2_ yield of 57.16%, and NbO_2_ had the highest 4e^−^ catalytic efficiency with n = 3.96 and a H_2_O_2_ yield of 2.18%, while the Co─Co_2_Nb_5_O_14‐x_@PCNFs showed a n value of 3.56 and a H_2_O_2_ yield of 22.00%, confirming that the Nb^4+^ in Co─Co_2_Nb_5_O_14‐x_@PCNFs had a notable promotional effect on 4e^−^ catalysis. The long‐term ORR stability was determined by the chronoamperometry at 0.6 V Vs. RHE (Figure 3d; Figure S23, Supporting Information). After 30 000 s, the current retention of the Co─Co_2_Nb_5_O_14‐x_@ PCNFs was 93.45%, which was only lower than that of PCNFs (98.30%) but much higher than the commercial catalyst (87.39%), Co@PCNFs (81.56%), NbO_2_@PCNFs (88.31%), and Nb_2_O_5_@PCNFs (78.09%), confirming its excellent durability.

The OER performance was also evaluated using a three‐electrode system, with the catalyst coated onto a carbon paper. Following the LSV detection, a plethora of air bubbles overflowed on the carbon paper, showcasing the high OER activity (Figure S24, Supporting Information). As indicated in Figure 3e and Figure S25 (Supporting Information), the overpotential of Co─Co_2_Nb_5_O_14‐x_@PCNFs was 296.3 mV at 10 mA·cm^−2^, superior to Co@PCNFs (327.3 mV), the commercial catalyst (308.3 mV), NbO_2_@PCNFs (319.3 mV), Nb_2_O_5_@PCNFs (474.3 mV), and PCNFs (578.3 mV). The low overpotential of NbO_2_@PCNFs indicated that Nb^4+^ served as the main OER active site in Co─Co_2_Nb_5_O_14‐x_@ PCNFs. The LSV curve of the blank carbon paper was also tested to eliminate background errors (Figure S26, Supporting Information). The Tafel curves of these catalysts are shown in Figure 3f. The Co─Co_2_Nb_5_O_14‐x_@PCNFs delivered a low Tafel slope of 115 mV·dec^−1^, which was close to the commercial catalyst (108 mV·dec^−1^) and Co@PCNFs (117 mV·dec^−1^), but superior to PCNFs (276 mV·dec^−1^), NbO_2_@PCNFs (167 mV·dec^−1^), and Nb_2_O_5_@PCNFs (193 mV·dec^−1^), indicating the high OER kinetics. The long‐term OER stability was determined by the chronoamperometry at 1.6 V Vs. RHE (Figure 3g; Figure S27, Supporting Information). After 30 000 s, the current retention of Co─Co_2_Nb_5_O_14‐x_@ PCNFs was 93.03%, surpassing the commercial catalyst (85.67%), Co@PCNFs (81.16%), NbO_2_@PCNFs (88.05%), Nb_2_O_5_@PCNFs (75.76%), and only trailing PCNF (98.40%). As shown in Figure 3h and Figure S28 (Supporting Information), the Co─Co_2_Nb_5_O_14‐x_@PCNFs had the smallest bifunctional indicator of ORR and OER (ΔE = 0.69), outperforming the commercial catalyst (ΔE = 0.71), as well as other control catalysts. The high bifunctional catalytic activity of the Co─Co_2_Nb_5_O_14‐x_@PCNFs surpassed most reported Co‐based catalysts, as shown in Figure 3i and Table S2 (Supporting Information).

### 2.4 DFT Calculations and In Situ Studies for Mechanistic Analysis

**Figure 4:**
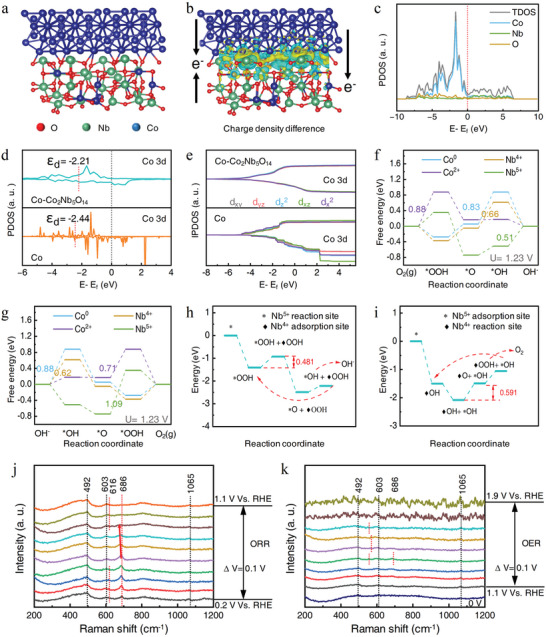
DFT calculations and catalytic mechanisms. a) Side view of the theoretical model of Co─Co_2_Nb_5_O_14_ and b) its charge density differences. Yellow and blue represent the accumulation and loss of charges, respectively. c) PDOS of the theoretical model of Co─Co_2_Nb_5_O_14_. d) PDOS and e) IPDOS of the two theoretical models. Gibbs free energy diagrams for four sites of Co─Co_2_Nb_5_O_14_ in f) ORR and g) OER. Optimal O‐species adsorption and migration pathways of the h) ORR and i) OER. In situ electrochemical Raman spectroscopy of the j) ORR and k) OER.

The specific active sites for distinct ORR/OER were further confirmed by DFT calculation. First, theoretical models were constructed for pure Co and lattice interlocked Co─Co_2_Nb_5_O_14_ (**Figure** **4**a; Figure S29, Supporting Information). To observe the interaction between Co and Co─Co_2_Nb_5_O_14_, the charge density difference for Co and Co─Co_2_Nb_5_O_14_ models was calculated (Figure 4b; Figure S30, Supporting Information). Both Co and Co─Co_2_Nb_5_O_14_ transferred electrons to their interface, and the overall electron transfer tendency was from Co to Co─Co_2_Nb_5_O_14_. The valence of Nb was determined based on the charge trends calculated from the Bader and DDEC6 charge analysis. The total density of states (DOS) and projected density of states (PDOS) were employed to analyze the electronic state distribution in two models. Both models exhibited DOS crossing the Fermi level (E‐E_f_ = 0), with overlapping conduction and valence bands, characteristic of typical metallic behavior (Figure S31, Supporting Information). Figure 4c depicts the PDOS diagram of the Co─Co_2_Nb_5_O_14_ model, where the peaks of Nb/Co atoms overlapped with O, confirming their respective bonding to O. The significant broadening near the Fermi level indicated strong interactions between these atoms, which facilitated electron transfer to adsorbed O‐species during the ORR process.^[^ ^32^, ^33^, ^34^ ^]^ Moreover, the presence of Nb 4d hybrid orbitals caused the d‐band center of Co (−2.44 eV) in Co─Co_2_Nb_5_O_14_ to shift closer to the Fermi level compared to metallic Co (−2.21 eV, Figure 4d). This shift promoted the transfer of ORR intermediates, thereby enhancing the catalytic activity.^[^ ^35^ ^]^ The integrated PDOS (IPDOS) results for the two models (Figure 4e; Figure S32, Supporting Information) indicated that the electrons in the Co 3d orbitals of Co─Co_2_Nb_5_O_14_ were closer to the same energy level, suggesting a lower filling degree of the eg orbitals, which was advantageous for the OER process.^[^ ^36^ ^]^

Next, the 4e^−^ ORR & OER pathways for active sites of Co^0^, Co^2+^, Nb^4+^, and Nb^5+^ were studied to explore the bifunctional catalysis mechanism. During ORR, the adsorption‐free energy at all sites exhibited a decreasing trend at U = 0 V (Figure S33a, Supporting Information), suggesting that the reaction was spontaneous. According to the rate‐determining step (RDS) at the potential U = 1.23 V, as shown in Figure 4f, the ORR activity of the four sites was ranked as Nb^5+^>Nb^4+^>Co^0^>Co^2+^, with their respective energy barriers being 0.51,0.66,0.83, and 0.88 eV. For OER, the free energy of these active sites exhibited an overall upward trend at U = 0 V (Figure S33b, Supporting Information). According to the RDS at U = 1.23 V, the OER activity was ranked as Nb^4+^>Co^2+^>Co^0^>Nb^5+^, with their respective energy barriers being 0.62, 0.71, 0.88, and 1.09 eV. This challenges the conventional understanding that low‐valence transition metals are generally used as active sites for ORR, and high‐valence metals in metal oxides usually exhibit high OER activity.^[^ ^37^, ^38^ ^]^ This result was due to the unique dynamic electron transfer and special O‐species adsorption/migration mechanisms in the Co─Co_2_Nb_5_O_14_ structure. Therefore, considering the simulation, the optimal O‐species adsorption and migration pathways were further calculated through frequency correction and elementary reaction calculations. As shown in Figure 4h, in ORR, Nb^5+^ is the main active site and Nb^4+^ is the adsorption site, Nb^4+^ adsorbs ·OOH at step 1 and transfers it to Nb^5+^ at step 3. In OER (Figure 4i), Nb^5+^ (adsorption site) adsorbs ·OH at step 1 and transfers it to Nb^4+^ (active site) at step 3. The energy barrier of the RDS for ORR and OER declined to 0.481 and 0.591 eV, respectively, due to this specific bifunctional mechanism (detailed in Figure S34, Supporting Information).

In situ electrochemical Raman spectroscopy (Figure S35, Supporting Information) was employed to elucidate the catalytic mechanism. Spectra were obtained by applying different bias voltages corresponding to the ORR/OER process under a 532 nm laser irradiation. As shown in Figure 4j,k, the peak near 1065 cm^−1^ corresponded to the stretching vibration of the MOO^−^ structure, which was an oxygen intermediate, indicating effective adsorption of the intermediates by the metal active sites of the catalyst.^[^ ^39^, ^40^ ^]^ During ORR, the two peaks near 616 and 686 cm^−1^ corresponded to the high wavenumber bands of Nb─O stretching vibrations.^[^ ^41^ ^]^ As the voltage increased, the peak at 616 cm^−1^ began to disappear at 0.6 V and reappeared at 0.9 V, while the peak at 686 cm^−1^ started to decrease at 0.6 V and disappeared at 1.0 V, indicating that the ORR process began at 0.6 V and intermediates were produced at these two peaks. The significant redshift of the peak at 686 cm^−1^ between 0.6 and 0.9 V suggested that this process was accompanied by Nb─O bond reconstruction. In the OER process, the peaks at 552, 565, and 686 cm^−1^ corresponded to Nb─O bonds.^[^ ^42^ ^]^ The peak at 552 cm^−1^ disappeared at 1.5 V and reappeared at 1.7 V, accompanied by presenting the 565 cm^−1^ peak in this process, indicating the dynamic transfer of oxygen intermediates among these two different Nb sites. The peak at 686 cm^−1^ appeared and then disappeared at 1.4 V, indicating the production of oxygen intermediates at this peak. Additionally, the peaks at 492 and 603 cm^−1^, corresponding to Co─O and CoOOH, respectively, showed no significant changes during ORR and OER.^[^ ^43^, ^44^ ^]^ These results indicated that the main active sites for ORR and OER were the Nb sites rather than Co, and there was a phenomenon of oxygen intermediates dynamic transferring among different Nb sites during the catalysis.

By combining theoretical simulation with the in situ Raman characterization, a unique dynamic electron transfer mechanism, and a bidirectional O‐species adsorption/migration mechanism were proposed. In ORR and OER, Nb^5+^ and Nb^4+^ served as the main active sites and adsorption auxiliary sites, respectively, while the bidirectional adsorption/migration promoted reversible reactions. Meanwhile, Co NDs functioned as powerful electronic regulation units for Co_2_Nb_5_O_14_ NRs. The electron transfer from Co to Co_2_Nb_5_O_14_ reduced the reaction energy barrier of ORR, and electron transfer from Co_2_Nb_5_O_14_ to interface avoided charge accumulation near the OER active sites, thus adapting the electronic structure to different needs of ORR and OER. The dynamic electron transfer and bidirectional adsorption‐migration mechanisms constitute a dynamically reversible catalytic interface, collectively enhancing the efficiency and stability of reversible ORR/OER.

### 2.5 Electrochemical Performance of Rechargeable Liquid Type ZABs

**Figure 5:**
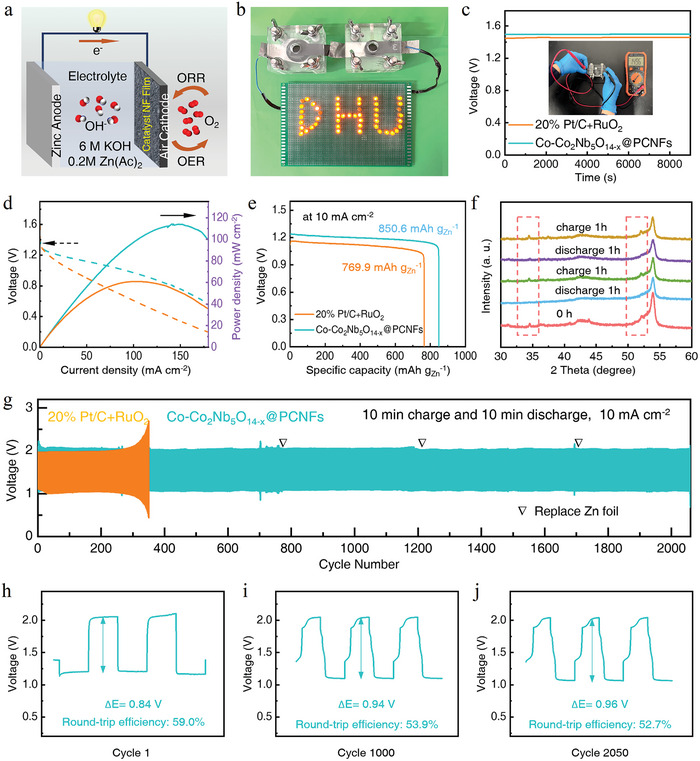
Electrochemical performance of rechargeable liquid type ZABs. a) Schematic diagram of a rechargeable liquid ZAB containing the Co─Co_2_Nb_5_O_14‐x_@PCNFs catalyst. b) Demonstration of using two ZABs to light the LED board. c) OCV plots of different ZABs. d) Discharge polarization curves and power densities of liquid ZABs. e) Discharge curves at 10 mA·cm^−2^ of liquid ZABs with different catalysts. f) XRD curves after discharging and charging. g) Long‐term cycling stability of two ZABs at 10 mA·cm^−2^. Voltage profiles of ZABs of h) cycle 1, i) cycle 1000, and j) cycle 2050.

To assess the application potential of the bifunctional electrocatalyst of Co─Co_2_Nb_5_O_14‐x_ @PCNFs, ZABs were assembled where the Co─Co_2_Nb_5_O_14‐x_@PCNFs served as the air cathode catalyst, and nickel foam composited carbon paper functioned as the cathode, complemented by a zinc foil acting as the anode (**Figure** **5**a; Figure S36, Supporting Information). As shown in Figure 5b and Figure S37 (Supporting Information), the assembled ZABs could light up the LED display board and light bulb. The electrochemical workstations and Land battery testing systems were used to measure the electrochemical performance (Figure S38, Supporting Information). As depicted in Figure 5c, the ZAB maintained a high open‐circuit voltage (OCV) of 1.496 V over 9000 s, which was close to that of the commercial catalyst based ZABs (1.458 V). From Figure 5d,e, both the power density (114.07 mW·cm^−2^) and specific capacity (850.6 mA h·g_Zn_^−1^) at 10 mA·cm^−2^ of the ZABs significantly surpassed that of the commercial catalyst‐based ZABs (66.08 mW·cm^−2^ and 769.9 mA h·g_Zn_^−1^).

From the charge–discharge curves (Figure 5g), it was observed that the as‐assembled ZABs could cycle stably for 2050 cycles at 10 mA·cm^−2^, which was much better than the commercial catalyst‐based ZABs. In addition, the ZAB exhibited a small charge–discharge voltage gap of 0.84 V (with a charge voltage of 2.05 V and a discharge voltage of 1.21 V) and a high round‐trip efficiency of 59.0% (Figure 5h). After 1000 cycles, the voltage gap increased to 0.94 V with a charge voltage of 2.04 V and a discharge voltage of 1.10 V) and the round‐trip efficiency decreased to 53.9% (Figure 5i). Even after 2050 cycles, it still maintained high stability with a small voltage gap of 0.96 V (with a charge voltage of 2.03 V and a discharge voltage of 1.07 V) and a high round‐trip efficiency of 52.7% (Figure 5j). Then, XRD was utilized to analyze the dynamic changes in Co─Co_2_Nb_5_O_14‐x_@PCNFs during cycling. The testing program was set as discharging for 1 h followed by charging for 1 h, and then repeated it once. The peak intensity at 34.6° corresponding to Co_2_Nb_5_O_14_ (101) and 51.9° to Co (200) decreased after discharging, and the intensities of the two peaks increased again after charging (Figure 5f). Figure S39 (Supporting Information) summarizes the dynamic changes in detail. The peak at 53.9° corresponded to the carbon paper substrate. The results confirmed reversible dynamic changes of the lattice interlocked structure during charging and discharging, which contributed to the long‐term cycling stability of ZABs.

Additionally, the electrochemical performance of ZABs with the Co@PCNFs catalyst was also examined (Figure S40, Supporting Information), along with the cycling stability comparison of ZABs with Co─Co_2_Nb_5_O_14‐x_@PCNFs, the commercial catalyst, and Co@PCNFs as catalysts at 2 mA·cm^−2^ (Figures S41–S43, Supporting Information). From the discharge curves at various current densities, the voltage plateaus of the three catalysts were similar at low current densities, but Co─Co_2_Nb_5_O_14‐x_@PCNFs showed a more stable and higher voltage than the commercial catalyst at high current densities. By comparison, the discharge voltage plateaus of Co@PCNFs were always maintained at the lowest (Figure S44, Supporting Information). These results confirmed that the Co─Co_2_Nb_5_O_14‐x_@PCNFs catalyst had the highest discharge capacity and the best cycling stability in ZAB applications. Moreover, the Co─Co_2_Nb_5_O_14‐x_@ PCNFs‐based ZABs exhibited better durability and higher capacity compared to some of the recently reported cobalt catalysts‐based ZABs, as listed in Table S3 (Supporting Information).

### 2.6 Electrochemical Performance of Rechargeable Solid Type ZABs

**Figure 6:**
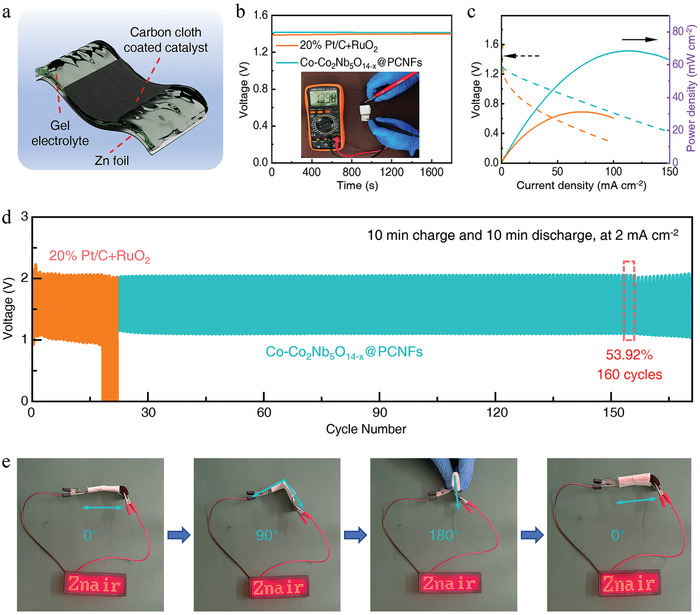
Electrochemical performance of solid ZABs. a) Schematic diagram of a solid ZAB structure. b) OCV plots of different ZABs. c) Discharge polarization curve and power density of solid ZABs. d) Long‐term cycling stability of different solid ZABs at 2 mA·cm^−2^. e) Demonstration of using a flexible ZAB to light up the LED display.

Solid ZABs utilizing the Co─Co_2_Nb_5_O_14‐x_@PCNFs catalyst were also developed, and the battery structure was shown in **Figures** **6**a and S45 (Supporting Information). In this structure, a carbon cloth coated with Co─Co_2_Nb_5_O_14‐x_@PCNFs was used as the air cathode, alkaline polyvinyl alcohol (PVA) gel as the electrolyte, and Zn foil as the anode. The commercial catalysts‐based solid ZABs were also prepared using the same method as controls. The loading capacity of catalysts was 1 mg·cm^−2^. The OCV of Co─Co_2_Nb_5_O_14‐x_@PCNFs and the commercial catalyst‐based ZABs were 1.398 and 1.327 V, respectively (Figure 6b; Figure S46a, Supporting Information). The peak power density calculated by discharge polarization curves of Co─Co_2_Nb_5_O_14‐x_@PCNFs was 68.4 mW·cm^−2^, surpassing the 31.3 mW·cm^−2^ achieved with commercial catalyst (Figure 6c). As shown in Figure S46b (Supporting Information), the discharge voltage of Co─Co_2_Nb_5_O_14‐x_@PCNFs gradually decreased from 1.24 to 1.00 V (the average value of each segment) with the current density varying from 0.5 to 10 mA·cm^−2^, which was superior to that of commercial catalysts (from 1.24 to 0.78 V).

As shown in Figure 6d, the solid ZABs exhibited high charge–discharge stability at 2 mA·cm^−1^, with negligible performance degradation even after 160 cycles. The ZABs showed a low polarization of 0.94 V (with charge and discharge voltages of 2.04 and 1.10 V, respectively), and the round‐trip efficiency was 53.92%. However, the solid ZABs using commercial catalysts could only be cycled 19 times, with a high polarization of 1.09 V and a low round‐trip efficiency of 47.09%. In short, the Co─Co_2_Nb_5_O_14‐x_@PCNFs showed an OCV close to that of commercial catalyst but exhibited higher power density and longer‐term cycling stability. Furthermore, flexible solid‐state ZABs under various bending angles (0°, 90°, 180°, unfolded) were also developed, as shown in Figure 6e and Figure S47a (Supporting Information). The bent ZAB could effortlessly illuminate the LED displaying “Zn‐air” and sustained the LED's illumination (Video S1, Supporting Information). Remarkably, the solid ZAB showed consistent performance across a bending range from 0 to 180°, with negligible variations in voltage gap and round‐trip efficiency (Figure S47b, Supporting Information).

## 3 Discussion

In this work, we have synthesized a stable and efficient bifunctional electrocatalyst of Co─Co_2_Nb_5_O_14‐x_@PCNFs with a unique dynamic reversible catalytic interface. Based on this catalyst, we have proposed a bidirectional adsorption/migration mechanism of oxygen species between the active sites of Nb^5+^ and Nb^4+^. Different from previous studies that focused on the active metal sites based on the metal‐support mechanism, we comprehensively explored the dynamic structural refactoring of Nb^5+^ and Nb^4+^ on the reducible oxides during ORR/OER. The bidirectional adsorption/migration of intermediates between Nb^5+^ and Nb^4+^ facilitated stable and reversible cyclic catalysis. In addition, we proposed a dynamic electron transfer mechanism between Co and Co_2_Nb_5_O_14_ at the SMSI interface, which promoted charge redistribution to satisfy different needs of ORR and OER, and reduced the energy barrier. The electron transfer from Co to Co_2_Nb_5_O_14_ provided sufficient electronics for the ORR active sites of Nb^5+^, and the electron transfer from Co_2_Nb_5_O_14_ to the SMSI interface avoided electron accumulations around the OER active sites of Nb^4+^. Therefore, the dynamic electron transfer redistributed the local charge density to adapt to the different needs of ORR and OER.

Moreover, the oxygen vacancy and oxygen‐enriched PCNF framework enhanced the adsorption and diffusion of intermediates, protected the active sites from rapid deactivation, and facilitated electron transfer during ORR and OER, thereby further improving catalytic activity, efficiency, and long‐cycle stability. Different from the traditional understanding that TMs mainly serve as the ORR active sites and TMOs serve as the OER active sites in TMs/TMOs combinations, this work found that both the ORR and OER sites were originated from TMOs, while the Co metal existed as an electronic regulatory unit. This study reveals the dynamic changes of TMOs and clarifies the reconstruction mechanism of the dynamic reversible interfaces in TMs/TMOs catalysis, thus providing a different perspective to design bifunctional TMs/TMOs catalysts for sustainable Zn‐air batteries.

In summary, we have designed a new electrocatalyst material with a different catalysis mechanism from the previously well‐recognized catalysis mechanism. The dynamic reversible catalytic mechanism simultaneously improved the activity and stability of ORR and OER. With the catalytic strategy design, the bifunctional catalyst achieved fast kinetics and great cycling stability over 30 000 s. The liquid Zn‐air batteries exhibited superior longer‐term stability over 2050 cycles in comparison with the commercial catalyst of Pt/C‐RuO_2_. After 2050 cycles, the round‐trip efficiency was still as high as 52.7%. In addition, the solid‐state Zn‐air batteries also exhibited leading long cycling stability and surpassed most Co‐based catalysts tested under similar conditions, confirming the superior advantages of the as‐designed catalyst. This competitive battery performance suggests that Co─Co_2_Nb_5_O_14‐x_@PCNFs could serve as a viable alternative to noble‐metal electrocatalysts.

## Conflict of Interest

The authors declare no conflict of interest.

## Author Contributions

J.Y. conceived the project. S.C. conducted the experiments and characterizations. J.Y. and S.C. wrote this paper and all authors contributed to discussing and revising the paper.

## Supporting information



Supporting Information

Supplemental Video 1

## Data Availability

The data that support the findings of this study are available from the corresponding author upon reasonable request.
