# Supplementary material for: Constructing Stable Bifunctional Electrocatalyst of Co─Co2Nb5O14 with Reversible Interface Reconstitution Ability for Sustainable Zn‐Air Batteries
Source: Adv Sci (Weinh). 2025 Mar 11;12(17):2413796. doi: 10.1002/advs.202413796 (PMC12061240; doi:10.1002/advs.202413796)
Supplement: Supplementary file 1 — Supporting Information [file ADVS-12-2413796-s001.pdf]

ADVANCED SCIENCE
Open Access

# Supporting Information

for *Adv. Sci.*, DOI 10.1002/advs.202413796

Constructing Stable Bifunctional Electrocatalyst of Co—$Co_2Nb_5O_{14}$ with Reversible Interface Reconstitution Ability for Sustainable Zn-Air Batteries

*Shuo Chen, Liang Zhang, Zheng Liu, Yuehui Chen, Shouzhu Li, Yuanyuan Zhang, Junyu Chen and Jianhua Yan**

# Supporting Information

## Constructing Stable Bifunctional Electrocatalyst of Co-$Co_2Nb_5O_{14}$ with Reversible Interface Reconstitution Ability for Sustainable Zn-Air Batteries

Shuo Chen[1], Liang Zhang[1], Zheng Liu[1], Yuehui Chen[1], Shouzhu Li[2], Yuanyuan Zhang[3], Junyu Chen[4], Jianhua Yan[1,2] *

[1]*College of Textiles, Donghua University, Shanghai 201620, China*

[2]*Xinjiang Key Laboratory of New Energy and Energy Storage Technology, Xinjiang Institute of Technology, Akesu 843100, China*

[3]*College of Textiles & Clothing, Qingdao University, Qingdao, 266071, China*

[4]*College of Textiles & Garments, Hebei University of Science and Technology, Shijiazhuang, 050018, China*

***Corresponding Author**: Prof. Jianhua Yan (yanjianhua@dhu.edu.cn)

## Experimental Section

***Synthesis of Co-$Co_2Nb_5O_{14-x}$@PCNFs films***. First, PAN (Mw = 90 000, Macklin) and PVP (Mw = 1300 000, Aladdin) with a weight ratio of 2:1 was added in DMF and stirred at 25 ºC until forming a clear solution. Then, 0.1 mol/L of $Co(acac)_2$ was added into the solution and stirred to form a stable sol. Finally, 0.1 mol/L of $Nb(OEt)_5$ was added dropwise to the mixed sol and stirred to form a stable deep-red sol. Next, the sol was injected into several sterile syringes, and electrospinning was carried out at a high-voltage (20 kV) field. Then, the as-spun NF films were heat-treated in an air oven at 280 °C for 2 h followed by carbonization in 8% $H_2$/Ar at 900 °C for 2 h. This method was also applied to the preparation of PCNFs and Co@PCNFs. $NbO_2$@PCNFs was carbonized in 8% $H_2$/Ar at 950 °C, while $Nb_2O_5$@PCNFs was carbonized in $N_2$ at 800 °C.

***Material characterization***. The material morphologies were characterized using SEM (Hitachi S-4800) and TEM (JEM-2100F). EDS and SAED were measured using the TEM machine. The crystal and chemical structures were analyzed by XRD (Bruker D8 ADVANCE) and FTIR spectrometer (Nicolet 8700). The element valences were analyzed using XPS (Thermo SCIENTIFIC ESCALAB Xi+). LabRAM HR Evolution (λ = 532 nm) was used to detect Raman spectroscopy. TG was performed on the SDT Q600 TG-DSC synchronous analyzer (TA Instruments Waters LLC, USA) with a heating rate of 10 $°C\cdot min^{-1}$. The absorption and desorption curves of $N_2$ were obtained using the Brunauer Emmet Teller analyzer (ASAP 2460, USA). The contents of Co and Nb in samples were analyzed using ICP (Agilent 5110).

***Electrochemical measurements for ORR/OER***. All the electrochemical measurements were carried out in a conventional three-electrode system on a CHI 660e electrochemical station (Shanghai Chenhua, China) at room temperature. The working electrode for ORR was a rotating disk electrode (RDE, S = 0196 $cm^2$, Pine Instrument Co, AFMSRCE 3699) made of glassy carbon coated with catalyst. During the testing process, the three-electrode system (saturated calomel electrode as reference electrode, glass carbon electrode as working electrode, and graphite electrode as the counter electrode) was utilized in 0.1 M KOH aqueous electrolyte that was bubbled with $O_2$ for 30 min to achieve the $O_2$ saturated solution. Preparation of the catalyst ink by mixing catalyst (5 mg) with isopropanol (600 μL), deionized water (360 μL), and 5% Nafion solution (40 μL) under ultrasound. Then, 30 μL of ink was dropped on the RDE and naturally dried in air. The cyclic

voltammetry (CV) was conducted at a scan rate of 50 $mV \cdot s^{-1}$, and the linear scanning voltammetry (LSV) was conducted at a scan rate of 5 $mV \cdot s^{-1}$. The working electrode for OER was a carbon paper (Toray, TGP-H-060) coated with catalyst. The ink preparation process for the catalyst was similar to that of ORR, with a loading amount of 1 $mg \cdot cm^{-1}$. During the testing process, the three-electrode system was utilized in 0.1 M KOH aqueous electrolyte that was bubbled with oxygen for about 30 min to achieve the $O_2$ saturated solution. During the i-t test, the catalyst was loaded onto a glassy carbon electrode and the measurement was conducted at a rotation speed of 400 rpm to eliminate the interference caused by bubble generation.

***DFT calculations***. All calculations were performed in the DFT framework with the projector augmented plane-wave method, as implemented in the Vienna ab initio simulation package.[1] The generalized gradient approximation proposed by Perdew, Burke, and Ernzerhof was selected for the exchange-correlation potential.[2] The cut-off energy for the plane wave was set to 500 eV. The energy criterion was set to $10^{-5}$ eV for the iterative solution of the Kohn-Sham equation. The recommended k-point path was generated using the post-processing VASPKIT package.[3] Due to the different amounts of atoms and sizes of the cells, the Brillouin zone was sampled using the Monkhorst–Pack method at a grid accuracy of 0.03 $2\pi$ $Å^{-1}$ for all systems.[4] A vacuum layer of 20 Å was added perpendicular to the sheet to avoid artificial interactions between periodic images. All the structures were relaxed until the residual forces on the atoms had decreased to less than 0.02 eV/Å. Full bulk relaxations, with atomic positions and cell (volume and shape) able to relax, were performed together using the conjugate gradient algorithm.[5] VESTA and VASPKIT were the programs used for modeling and data post-processing. Moreover, the change in Gibbs free energy (ΔG) of each species was obtained from the following equation: $\Delta G = \Delta E_{DFT} + \Delta E_{ZPE} - TS + \Delta G_U$, where $\Delta E_{DFT}$ is the electronic energy calculated from DFT calculations. Herein the value of $\Delta E_{ZPE}$- TS was set to be 0.4 for OOH, 0.05 for O, and 0.35 eV for OH intermediates, respectively.[6] The ΔGU was deduced from -neU, where n is the number of transferred electrons, and U is the electrode potential versus RHE (reversible hydrogen electrode).

***Assembly and measurements of Liquid ZABs***. The battery contained a Zn foil anode with a thickness of 0.05 mm (99.99%), an alkaline electrolyte containing 6.0 M KOH and 0.2 M $Zn(Ac)_2$, and an air cathode made of nickel foam composited carbon paper with a gas diffusion layer. The gas

diffusion layer with an area of 1.0 $cm^2$ allowed $O_2$ diffusion from air to the catalyst. The catalyst layer was made by drop-casting the catalyst ink onto the nickel foam with a loading of 1 $mg \cdot cm^{-2}$. ZABs fabricated using 20%Pt/C and $RuO_2$ (Sinero, SRu75) with a mass ratio of 1: 1 were used for comparison. The electrochemical workstation (CHI 660E) was used to test the voltage profiles and polarization curves, and the Land battery test system was used to measure cycle performance. Each cycle was set for 20 min (10 min discharge and 10 min charge).

**Assembly and measurements of solid ZABs**. The solid-state battery contained a Zn foil anode and an air cathode, which were connected face-to-face with the intermediate gel electrolyte (2 x 2 $cm^2$). It was encapsulated with breathable waterproof tape to ensure the smooth entry and exit of $O_2$. The electrolyte solution containing 10% PVA, 6.0 M KOH, and 0.2 M $Zn(Ac)_2$ was frozen at -20 °C for 2 h, and then thawed at room temperature to form a gel electrolyte. The air cathode was made of carbon cloth with the catalyst (Co-$Co_2Nb_5O_{14-x}$@PCNF or 20% Pt/C+$RuO_2$). All battery tests were conducted under an ambient atmosphere.

## Supporting Figures

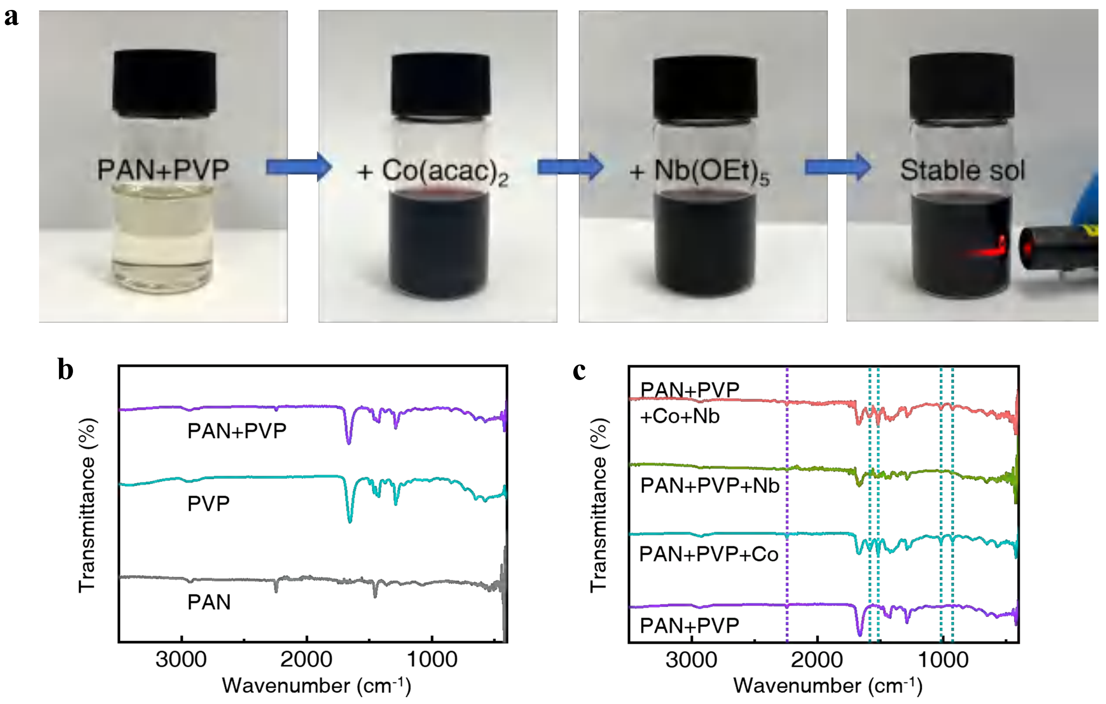


**Figure S1. Characteristics of stable sols**. (**a**) The general process of preparing stable sols for electrospinning. The final spinning fluid displayed a distinct Tyndall effect, indicating it was a stable polymer-metal sol. The deep purple color indicated the coordination between PAN and acetylacetone cobalt. (**b**) FTIR spectra of sols for different polymers. Compared with the pure PAN and PVP solutions, there were not new chemical bonds for the PAN/PVP mixture, indicating no cross-linking occurred between the two polymers. (**c**) FTIR spectra of sols for different components. the addition of acetylacetone cobalt introduced new peaks at 1584, 1518, 1015, and 925 $cm^{-1}$, and the addition of ethoxyniobium also introduced a new peak at 1518 $cm^{-1}$. The peak at 1518 $cm^{-1}$ was attributed to the telescopic vibration of -C=N, and the peak at 2245 $cm^{-1}$ was due to the -C≡N stretching vibration of the PAN.

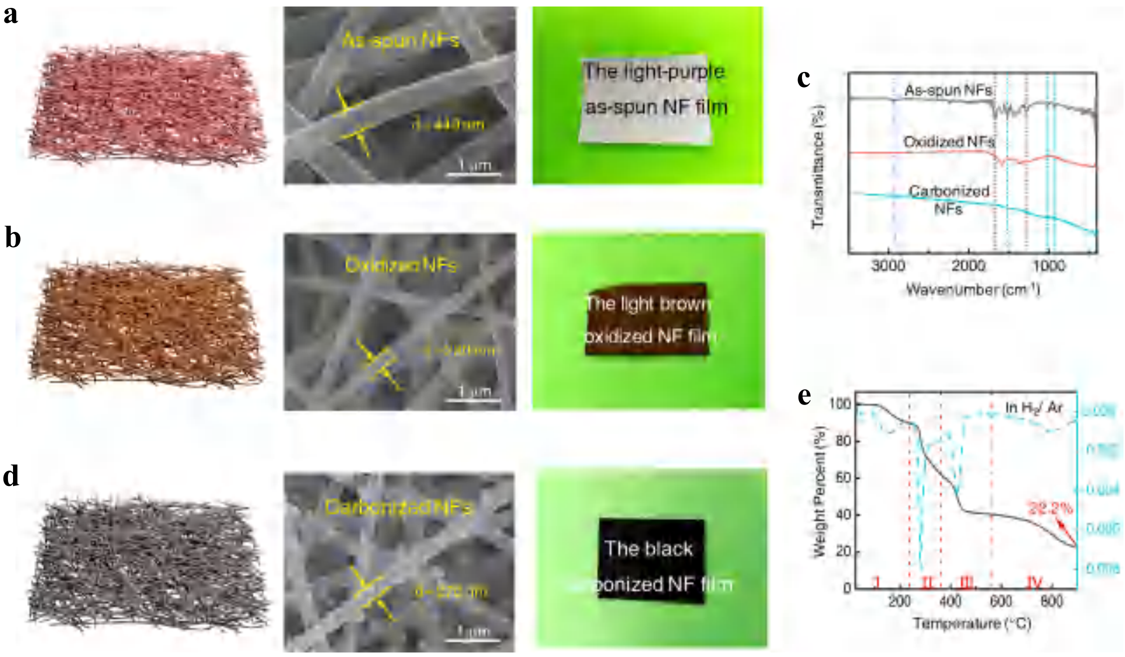


**Figure S2. Characteristics of material fabrication process**. Schematic diagram, SEM images, and digital photos of the (**a**) as-spun NFs, (**b**) oxidized NFs, and (**d**) carbonized NFs. (**c**) FTIR spectra of the as-spun NF films, oxidized NF films, and carbonized NFs. The disappearance of some functional groups during the high-temperature oxidation and carbonization indicated the formation of $Co_2Nb_5O_{14-x}$@PCNFs. (**e**) TG curve of the as-spun precursor NFs in $H_2$/Ar to express the calcination process from room temperature to 900 °C. The yield of the material was 22.2%.

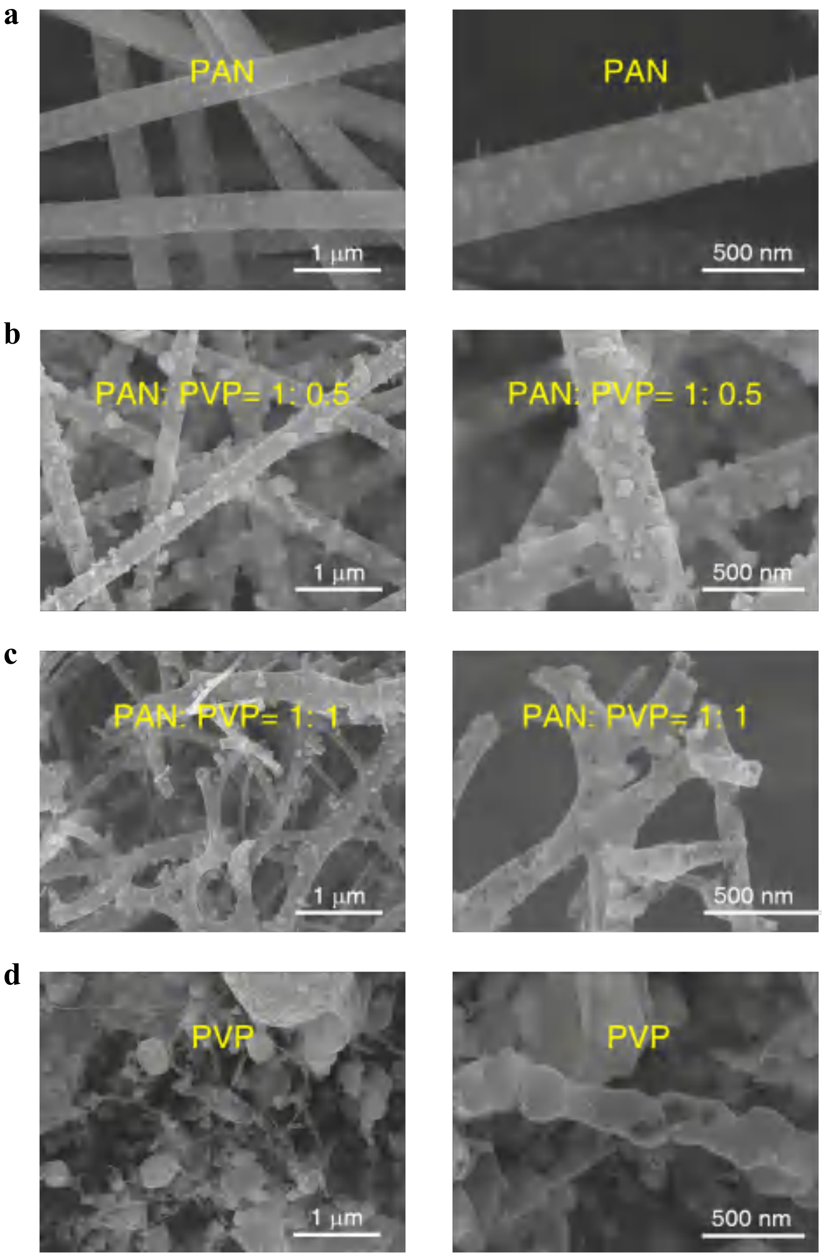


**Figure S3. SEM images of the electrospun NFs with different ratios of PAN and PVP**. (**a**) NFs only contained PAN. (**b**) NFs with a PAN and PVP ratio of 1: 0.5 (used in this work). (**c**) NFs with a PAN and PVP ratio of 1: 1. (**d**) NFs only contained PVP. PVP served as a pore regulator in the material forming process. The number of pores in the fibers gradually increased as the PVP content increased, but excessive PVP content would cause collapse of the NF structure.

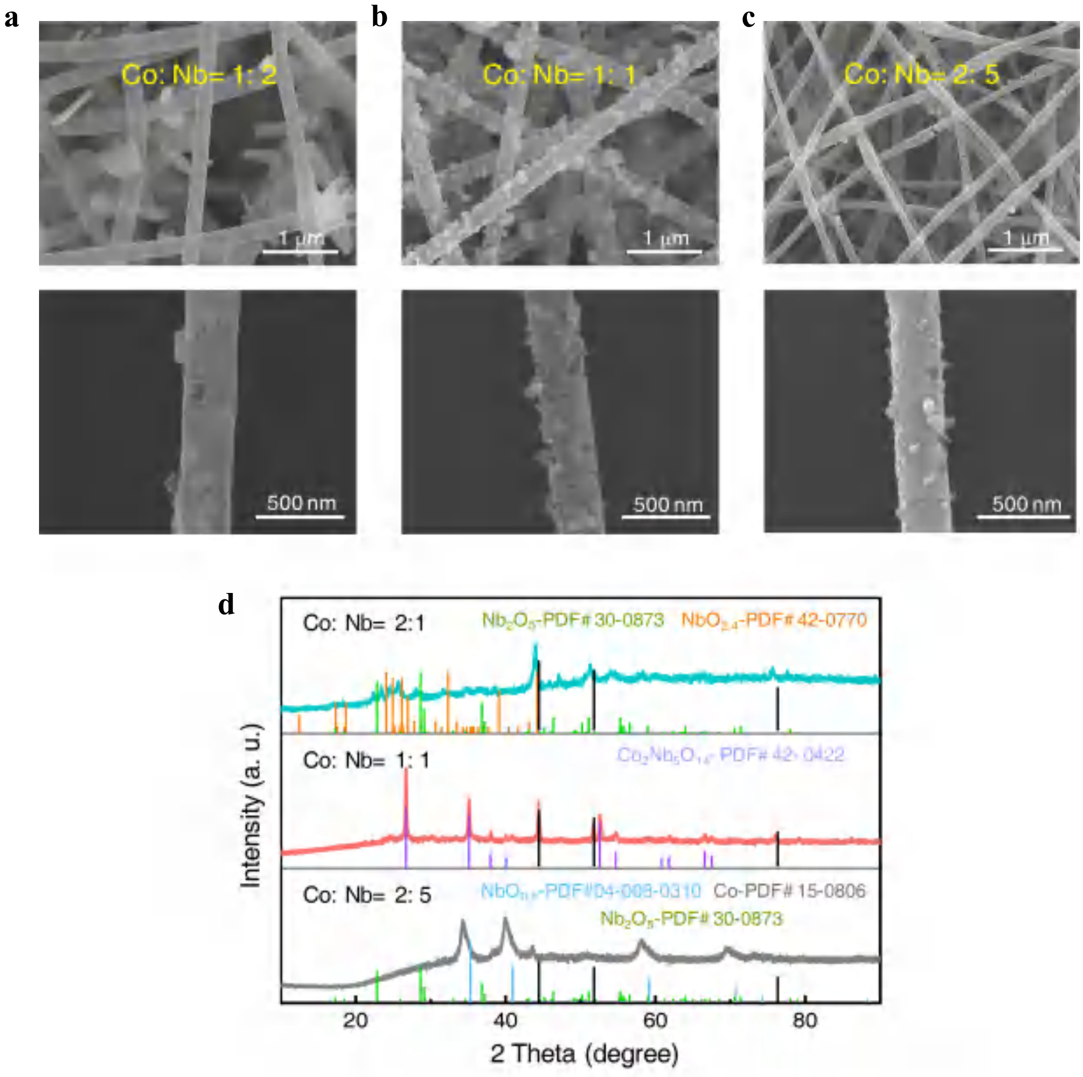


**Figure S4. Characteristics of the NFs with different ratios of Co and Nb**. SEM images of the NFs with a Co: Nb ratio of (**a**) 1: 2, (**b**) 1: 1, and (**c**) 2: 5. (**d**) XRD spectra and phase analysis of NFs with different ratios of Co and Nb. The XRD results confirmed that Co atom occupation at the $Nb^{5+}$ position resulted in the generation of numerous surface vacancies ($O_{vac}$) and $Nb^{4+}$.

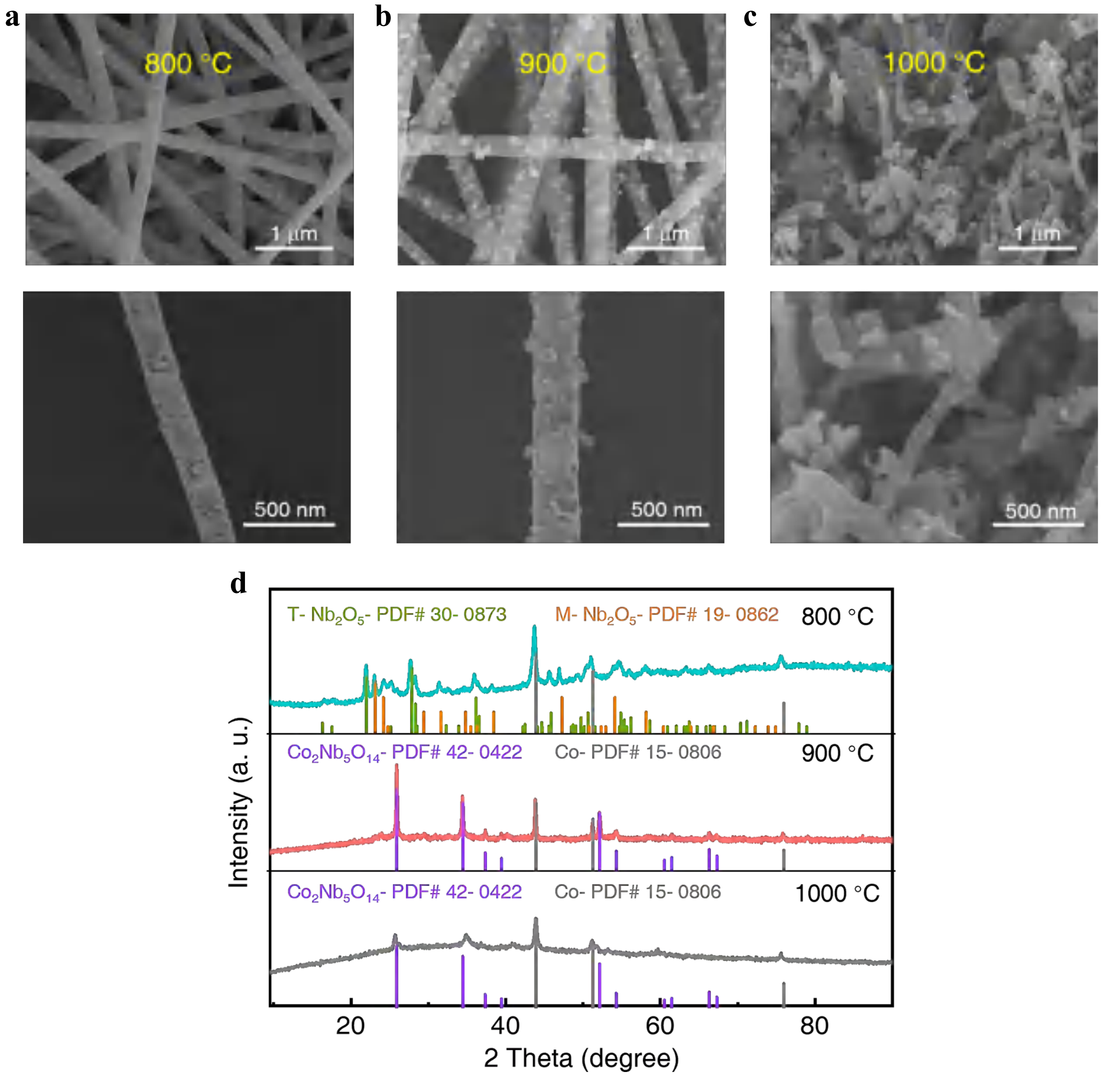


**Figure S5. Characteristics of NFs with different carbonization temperatures**. SEM images of NFs carbonized in (**a**) 800 °C, (**b**) 900 °C (This work), and (**c**) 1000 °C. (**d**) XRD spectra and phase analysis of different NFs. The NFs contained T-$Nb_2O_5$, M-$Nb_2O_5$, and Co at 800 °C. The $Co_2Nb_5O_{14}$ phase appeared at 900 °C, but its peak intensity decreased greatly at 1000 °C since it was reduced to $NbO_2$.

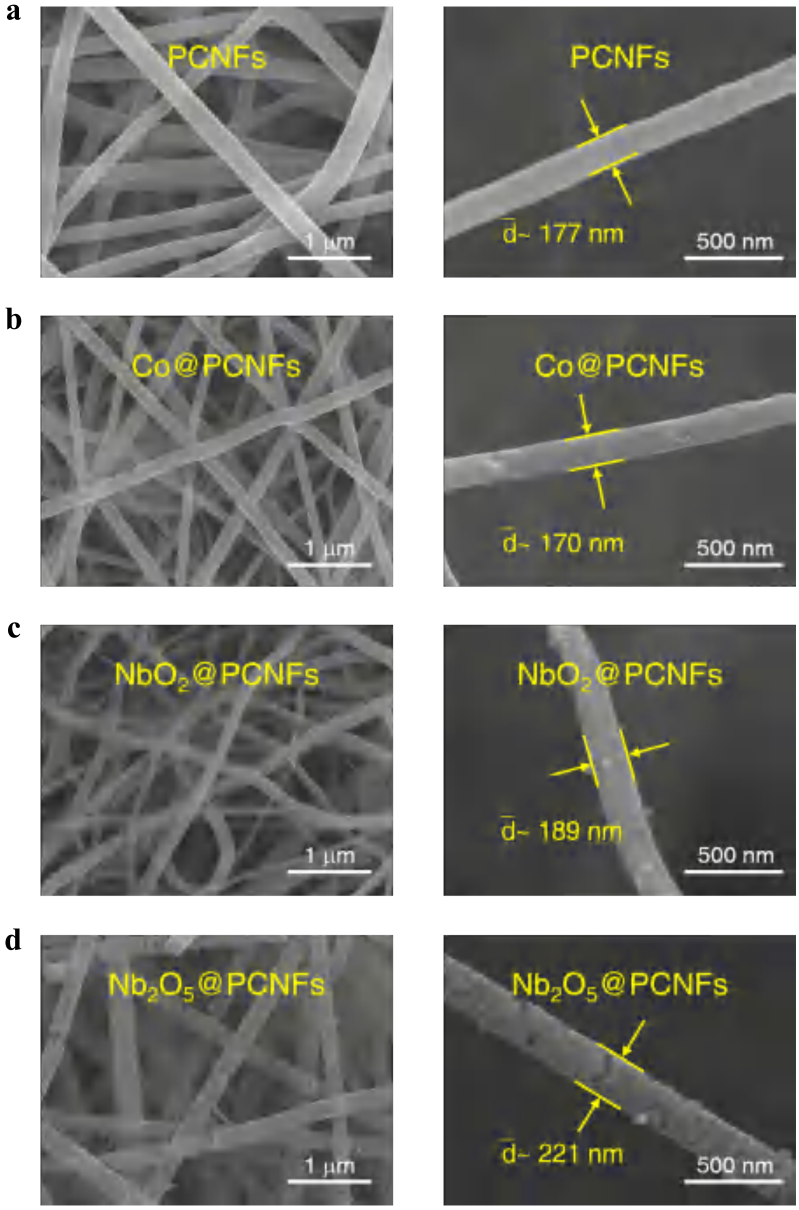


**Figure S6. SEM images** of (**a**) PCNFs, (**b**) Co@PCNFs, (**c**) $NbO_2$@PCNFs, and (**d**) $Nb_2O_5$@PCNFs. The average diameters of the four NFs were calculated as 177, 170, 189, and 221 nm, respectively.

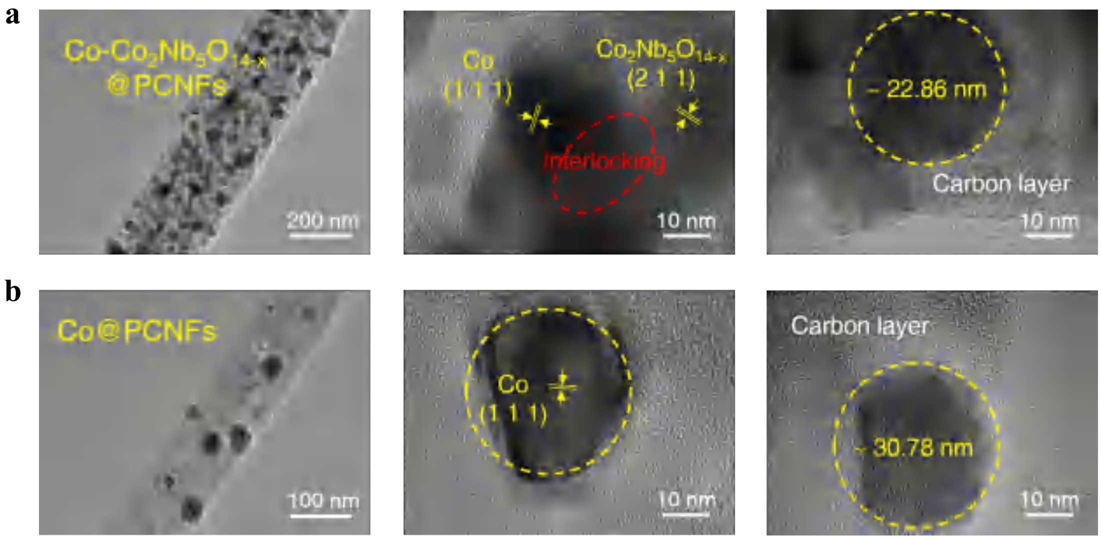


**Figure S7. TEM images** of (**a**) Co-$Co_2Nb_5O_{14-x}$@PCNFs and (**b**) Co@PCNFs. In Co-$Co_2Nb_5O_{14-x}$@PCNFs, Co NDs and $Co_2Nb_5O_{14}$ NRs were evenly dispersed and formed a distinct lattice interlocked SMSI structure. The average size of Co NDs in Co-$Co_2Nb_5O_{14-x}$@PCNFs was ~ 22.86 nm, which was smaller than that in Co@PCNFs (~ 30.78 nm).

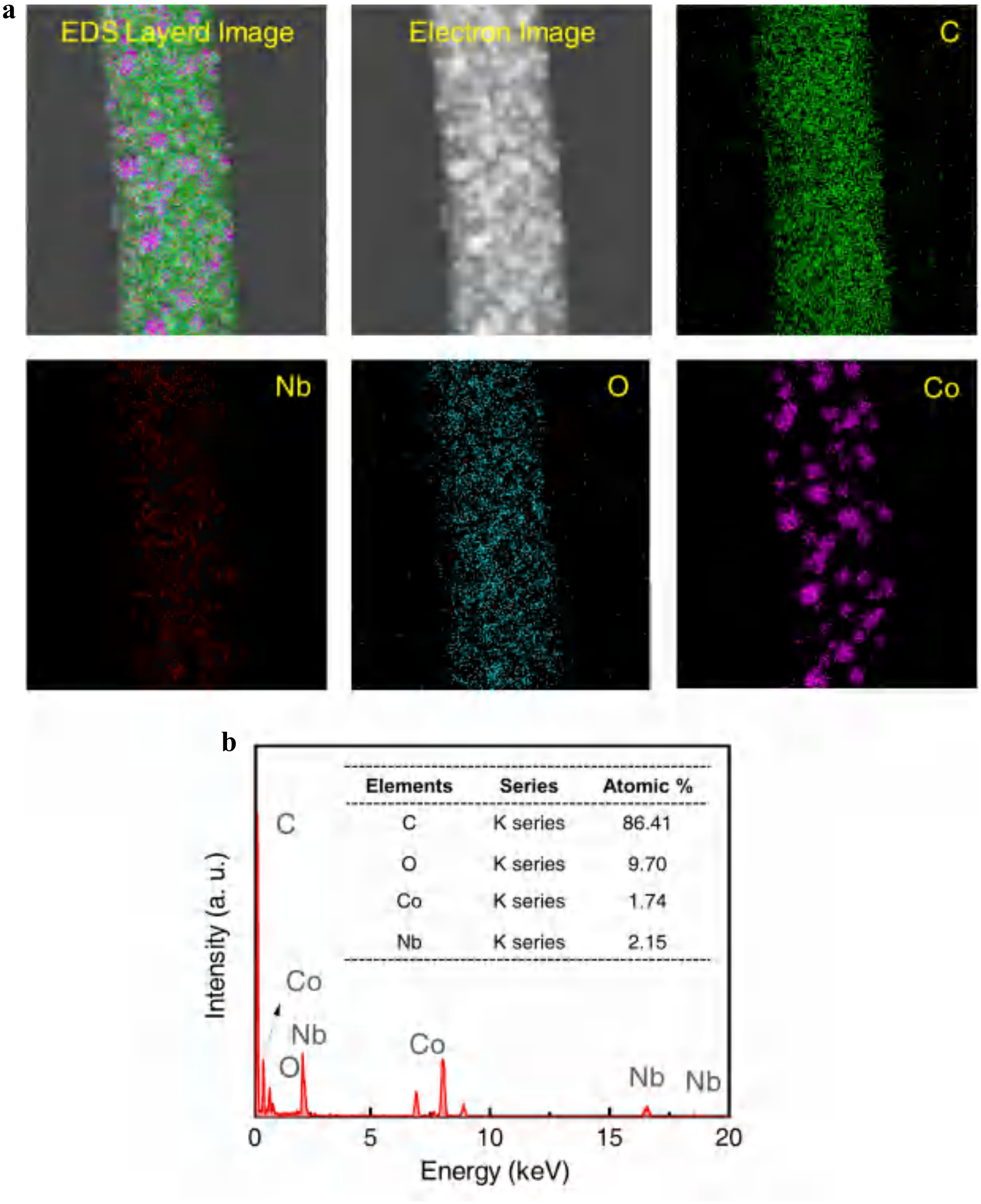


**Figure S8. EDS mapping of a single Co-$Co_2Nb_5O_{14-x}$@PCNF.** (**a**) The elements are evenly distributed in the PCNF. (**b**) Map sum spectrum. The atomic ratios of Co and Nb are 1.74% and 2.15%, respectively, which is close to 1:1.

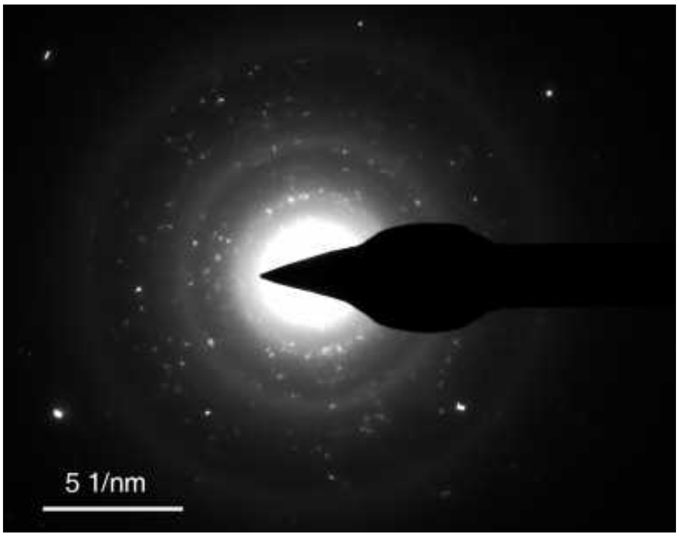


**Figure S9. Selected area electron diffraction (SAED) of Co-$Co_2Nb_5O_{14-x}$@PCNFs**.

The presence of linear diffraction rings confirmed that the Co-$Co_2Nb_5O_{14-x}$ had a polycrystalline structure

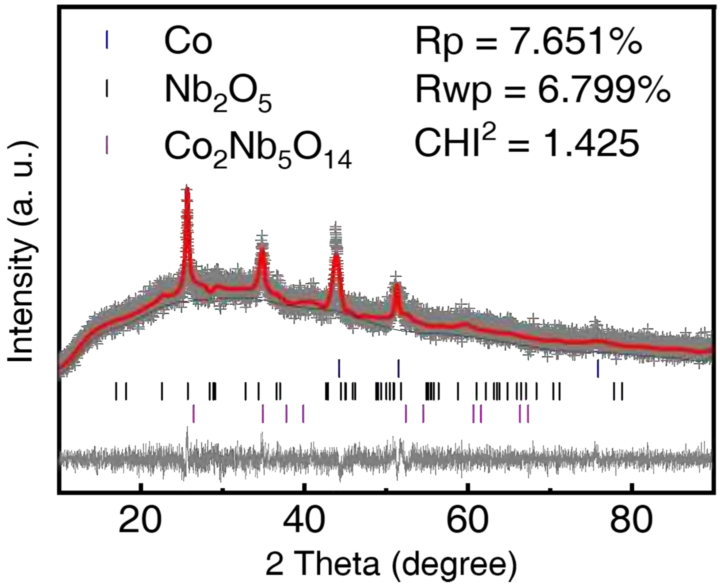


**Figure S10. XRD refined spectrum of Co-$Co_2Nb_5O_{14-x}$@PCNFs**. The step size for the measurement was 0.1 deg, and the measurement range was from 10° ~ 90°.The contents of Co, $Nb_2O_5$, and $Co_2Nb_5O_{14}$ were 55.79%, 14.57%, 29.64% respectively. The standard PDF cards were Co-PDF#15-0806, $Nb_2O_5$-PDF#30-0873, and $Co_2Nb_5O_{14}$-PDF# 42-0422.

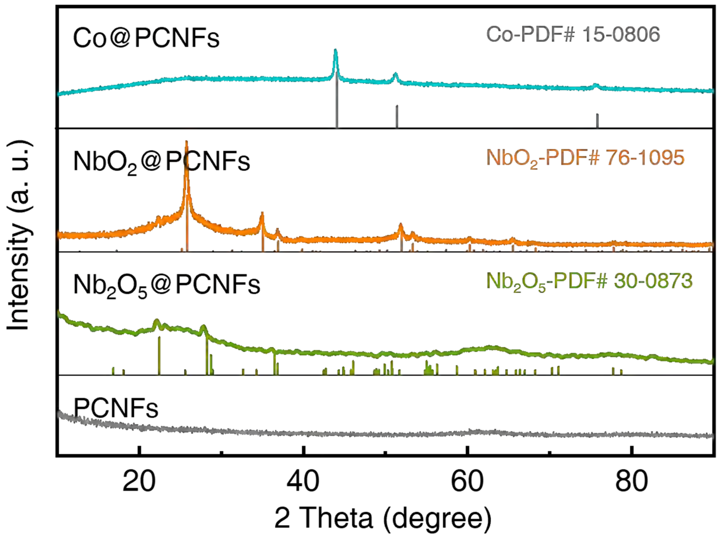


**Figure S11. XRD patterns of the four catalysts**. The step size for the measurement was 0.1 deg, and the measurement range was from 10° ~ 90°. The standard PDF cards of the three materials were Co-PDF#15-0806, $NbO_2$-PDF#76-1095, $Nb_2O_5$-PDF#30-0873, respectively. Because PCNFs is amorphous carbon without crystals, there is no corresponding PDF card.

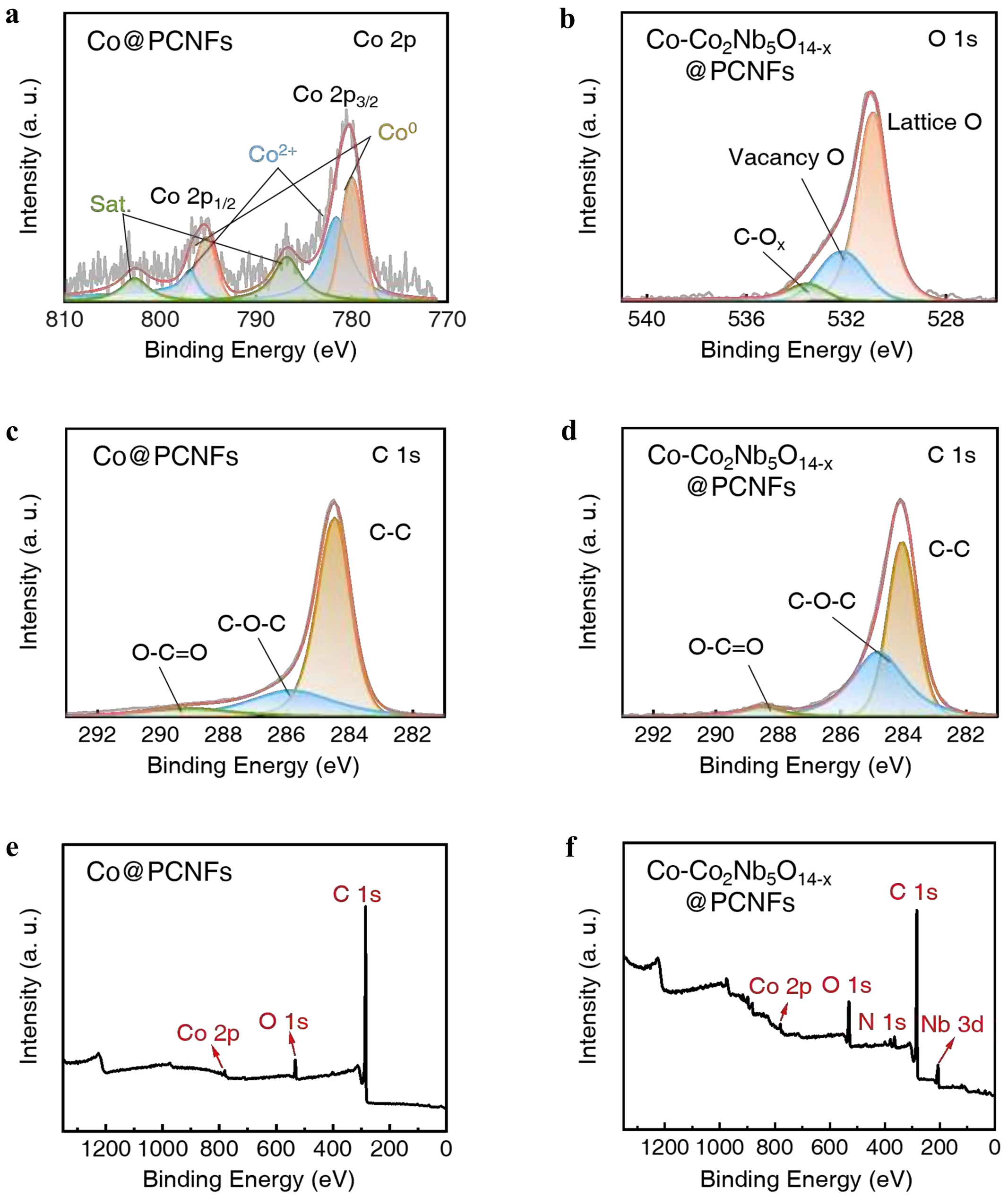


**Figure S12. XPS patterns of Co-$Co_2Nb_5O_{14-x}$@PCNFs and Co@PCNFs**. (**a**) Co 2p, (**c**) C 1s, and (**e**) full element spectrogram spectra of Co@PCNFs. (**b**) O 1s, (**d**) C 1s and (**f**) full element spectrogram of Co-$Co_2Nb_5O_{14-x}$@PCNFs.

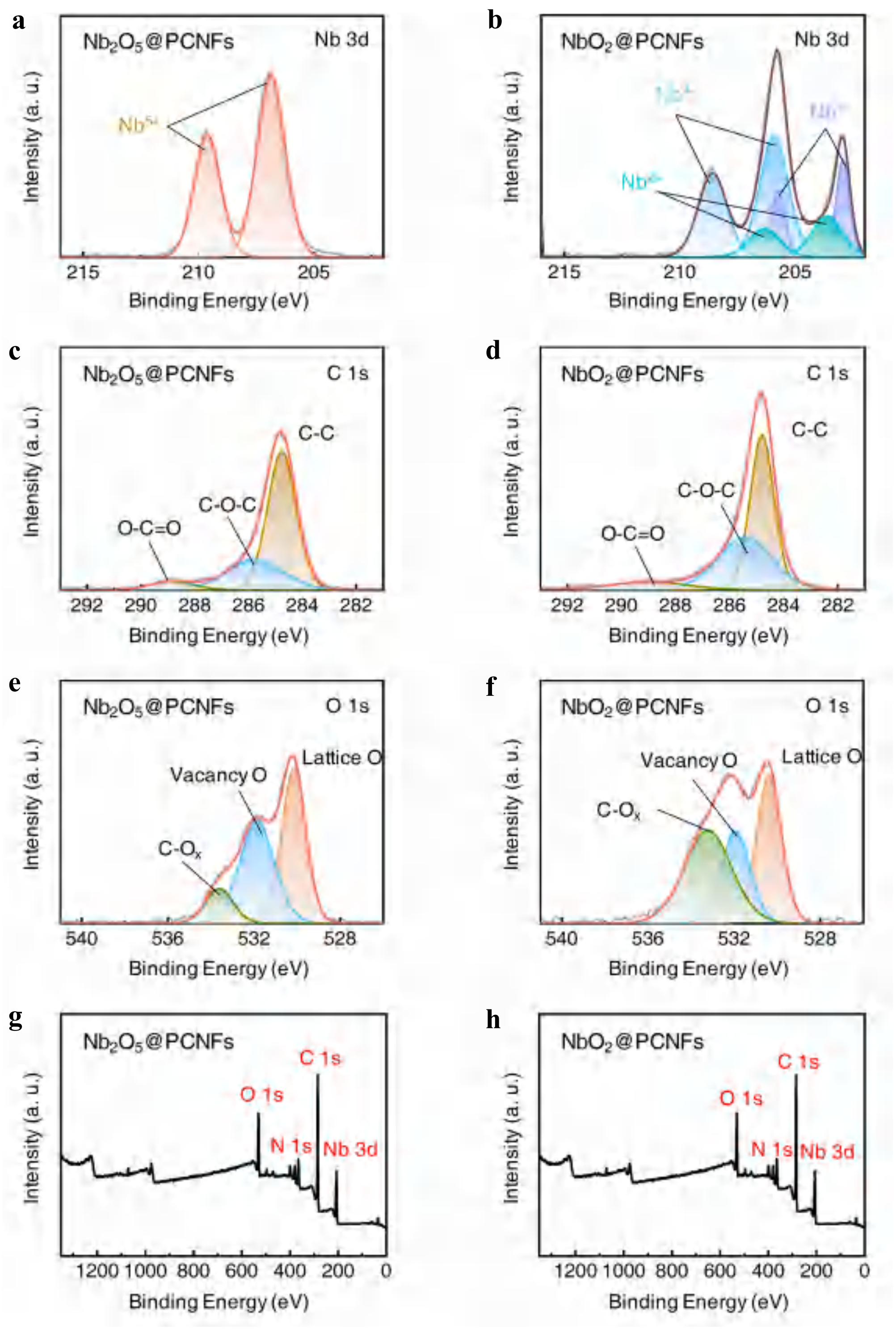


**Figure S13. XPS patterns of $Nb_2O_5$@PCNFs and $NbO_2$@PCNFs.** (**a**) Nb 3d, (**c**) C 1s, (**e**) O 1s and (**g**) full element spectrogram spectra of $Nb_2O_5$@PCNFs. (**b**) Nb 3d, (**d**) C 1s, (**f**) O 1s and (**h**) full element spectrogram spectra of $NbO_2$@PCNFs.

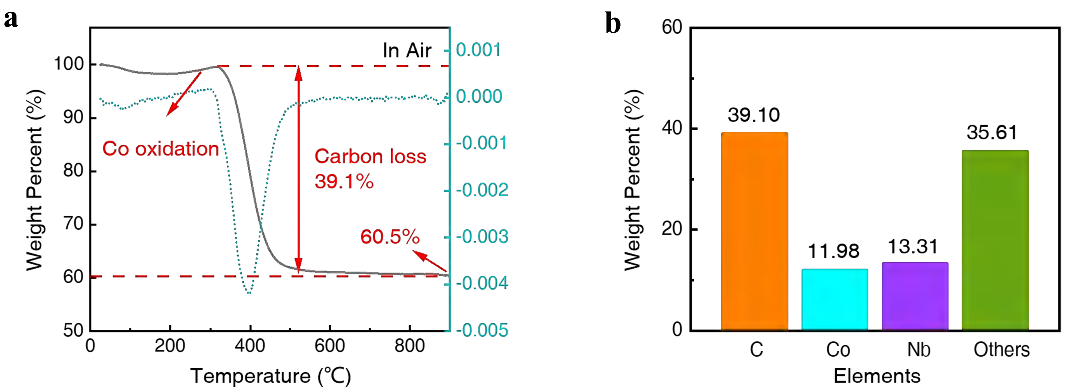


**Figure S14. The contents of elements in the Co-$Co_2Nb_5O_{14-x}$@PCNFs**. (**a**) TG curve of the Co-$Co_2Nb_5O_{14-x}$@PCNFs tested from room temperature to 900 °C. The weight increased in the temperature range of 130~323 °C was attributed to the oxidation of Co NDs. The C element lost rapidly in the range of 324~510 °C. The results showed that the C content was ~39.1 wt.%. (**b**) The content of Co and Nb tested by ICP was 11.98 w.t% and 13.31 wt.%, respectively. The content of other elements (35.61 wt.%) was drawn by combining the C content tested by TG. The detailed data of ICP was shown in Table S1.

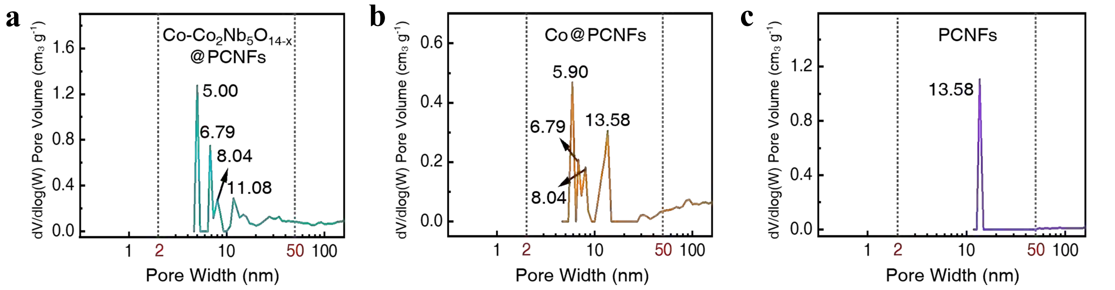


**Figure S15. Pore size distribution of the (a)** Co-$Co_2Nb_5O_{14-x}$@PCNFs, (**b**) Co@PCNFs, and (**c**) PCNFs. The pores of all three materials were concentrated in the range of 2~ 50 nm and were classified as mesopores. According to the pore volume size, it was sorted as Co-$Co_2Nb_5O_{14-x}$@PCNFs> Co@PCNFs> PCNFs.

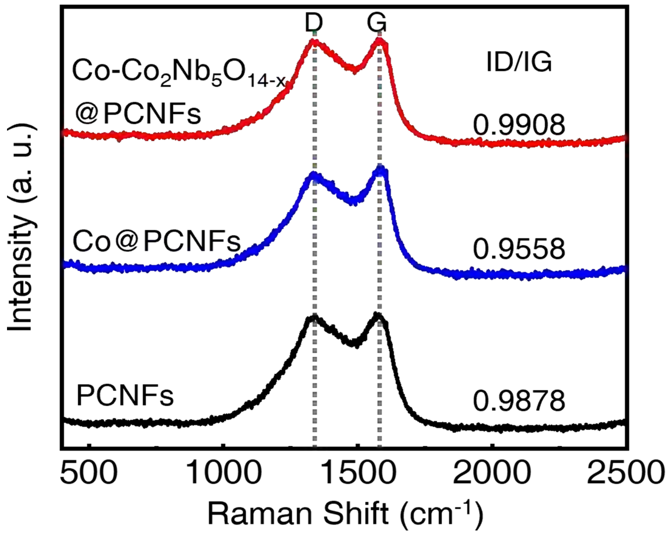


**Figure S16. Raman spectra of different materials**. The D-band at 1338 $cm^{-1}$ is associated with defects and disorders in carbonaceous materials, and the G-band at 1580 $cm^{-1}$ is associated with in-plane stretching vibration of $sp^2$-hybridized carbon.

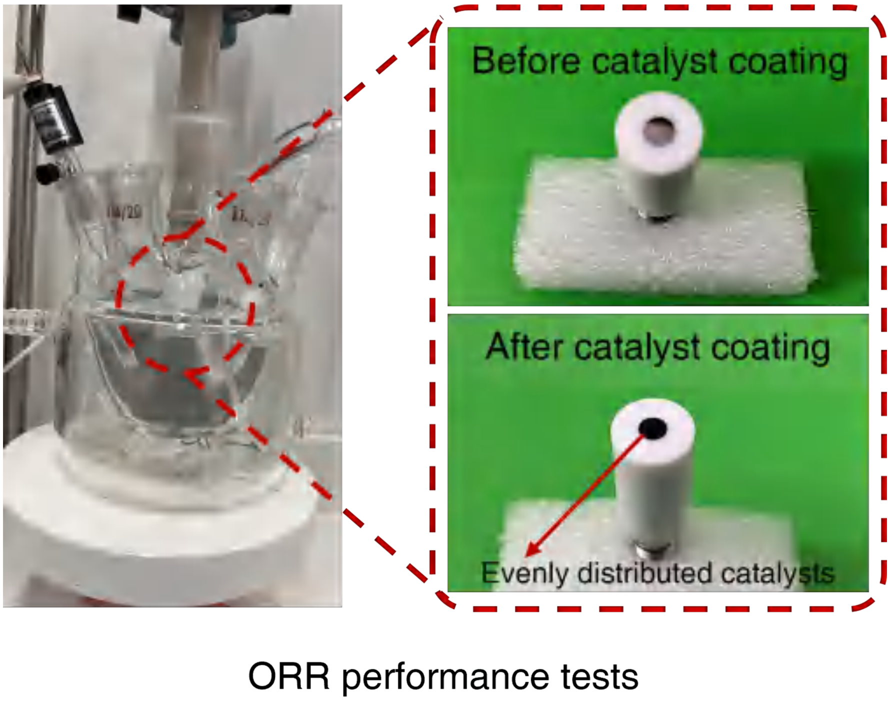


**Figure S17. ORR testing process**. The three-electrode test was performed using a Chenhua electrochemical workstation and a rotating disc electrode (RDE). The catalyst was uniformly coated on the smooth surface of the glassy carbon electrode.

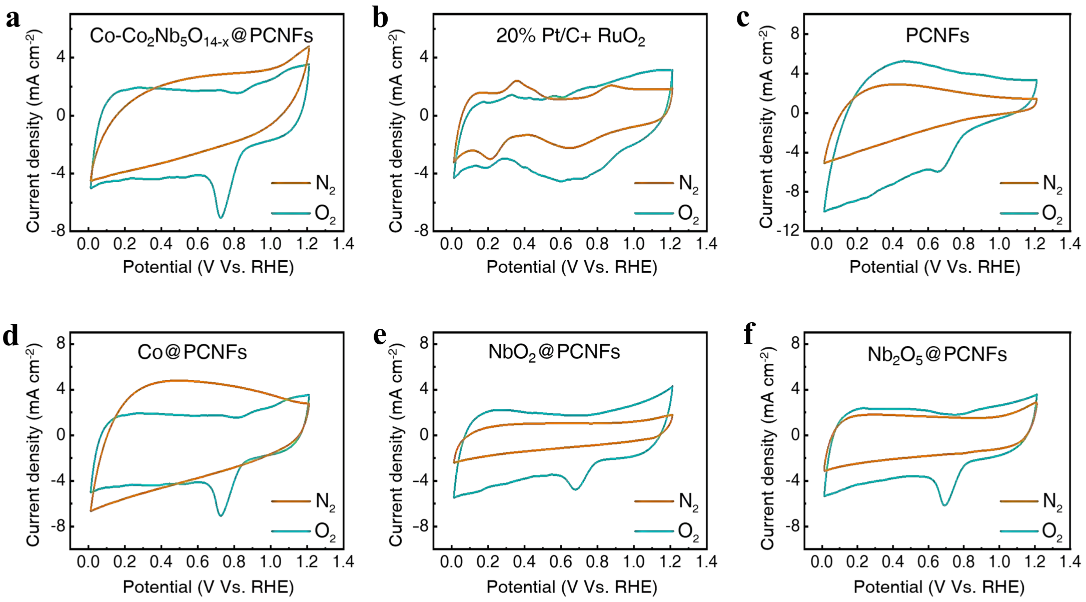


**Figure S18. CV curves of different catalysts tested in $N_2$ or $O_2$ atmospheres**. (**a**) Co-$Co_2Nb_5O_{14-x}$@PCNFs, (**b**) 20%Pt/C+ $RuO_2$, (**c**) PCNFs, (**d**) Co@PCNFs, (**e**) $NbO_2$@PCNFs, and (**f**) $Nb_2O_5$@PCNFs. The scan rate was 50 $mV \cdot s^{-1}$. Compared to $N_2$, all catalysts exhibited distinct reduction peaks in $O_2$, indicating that they all possessed ORR catalytic activity.

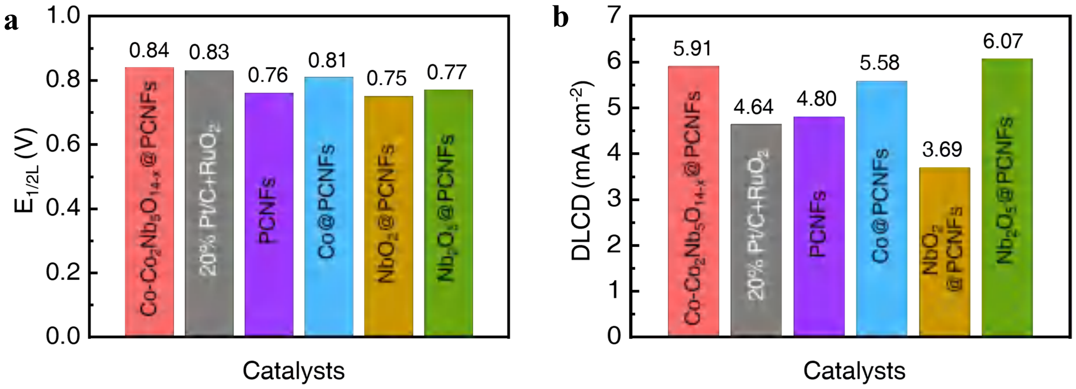


**Figure S19. $E_{1/2}$ and DLCD of the six catalysts for better comparison**. (**a**) $E_{1/2}$ of the six catalysts. (**b**) DLCD of the six catalysts. The data were obtained by subtracting the background current from the LSV curve measured at 1600 rpm. Larger $E_{1/2}$ and DLCD indicate better ORR catalytic activity.

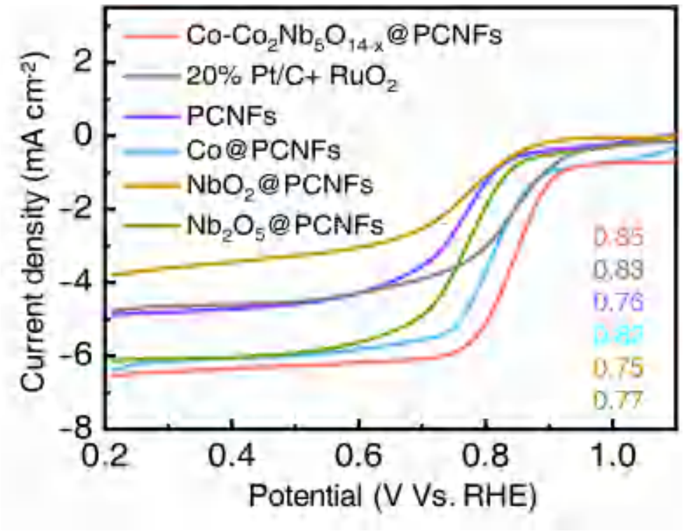


**Figure S20. LSV curves tested at 1600 rpm without deducting the background current**. The scan rate was 5 mV·s$^{-1}$. $E_{1/2}$ is the voltage corresponding to half of the diffusion-limited current density (DLCD).

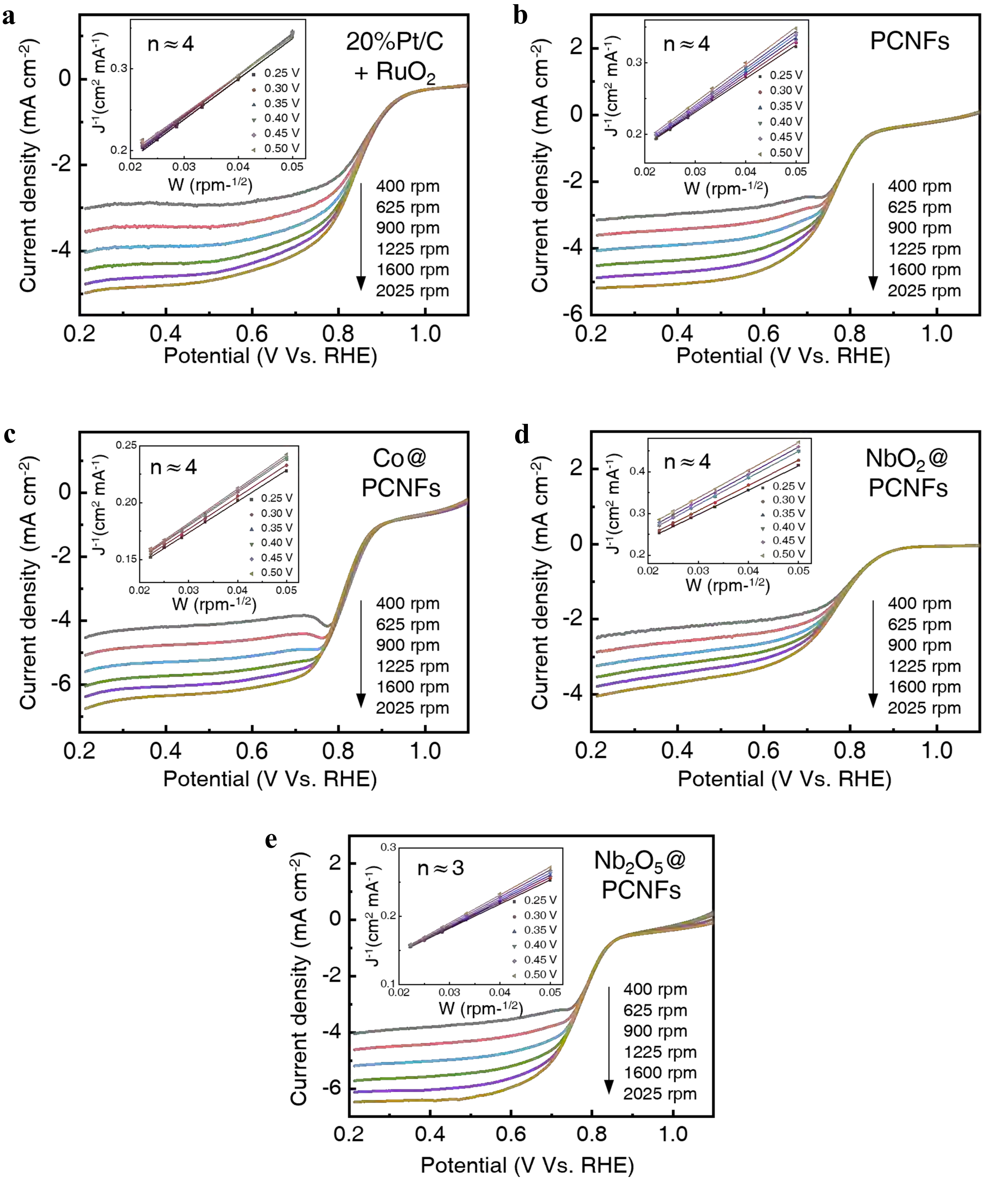


**Figure S21. LSV curves of different catalysts tested at different rotation speeds**. (**a**) 20%Pt/C+ $RuO_2$, (**b**) PCNFs, (**c**) Co@PCNFs, (**d**) $NbO_2$@PCNFs, and (**e**) $Nb_2O_5$@ PCNFs. The scan rate was 5 mV·s$^{-1}$. The inset figure was the K−L plot at different potentials. The K-L linear fitting lines of $NbO_2$@PCNFs could not coincide. The electron transfer number (n) for these catalysts were calculated to be close to 4, excepted $Nb_2O_5$@PCNFs (n ≈ 3).

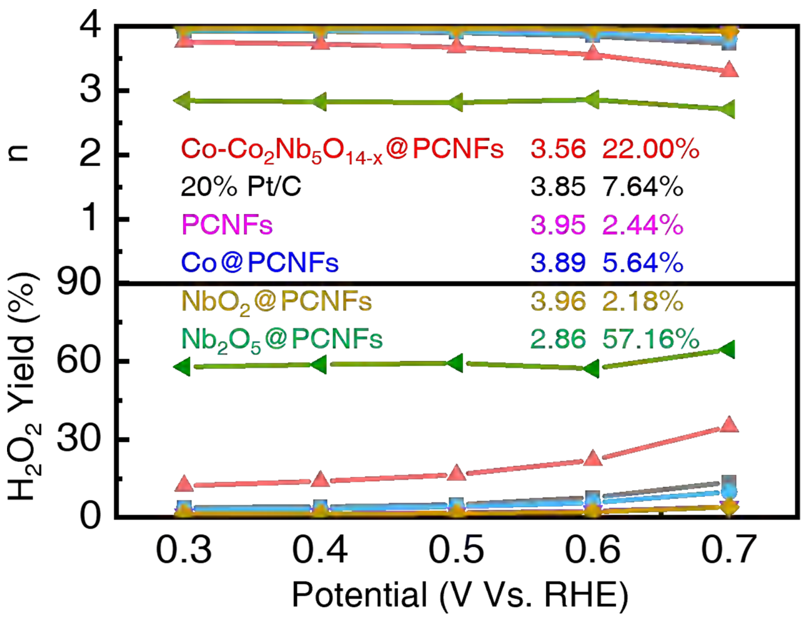


**Figure S22. Number of electron transfers (n) and $H_2O_2$ yield (%) calculated by RRDE tests**. The data at the potential of 0.6 V Vs. RHE were collected for comparison. The higher the n-value, the lower the $H_2O_2$ yield, and the higher the $4e^-$ catalytic efficiency.

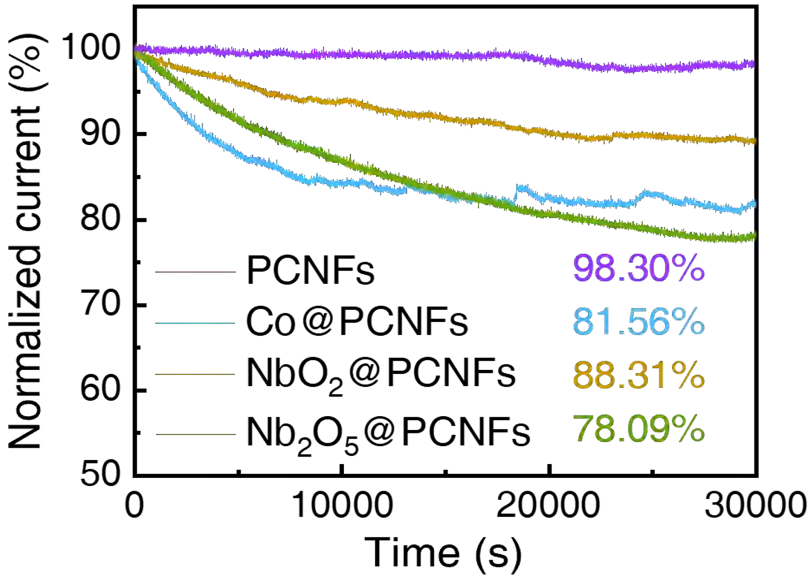


**Figure S23. i-t curves**. ORR electrocatalytic stability testing of the catalysts by chronoamperometry at 0.6 V Vs. RHE. After 30000 s, the normalized currents remained 98.3% (PCNFs), 81.56% (Co@PCNFs), 88.31% ($NbO_2$@PCNFs), and 78.09% ($Nb_2O_5$@PCNFs) of their initial currents, respectively.

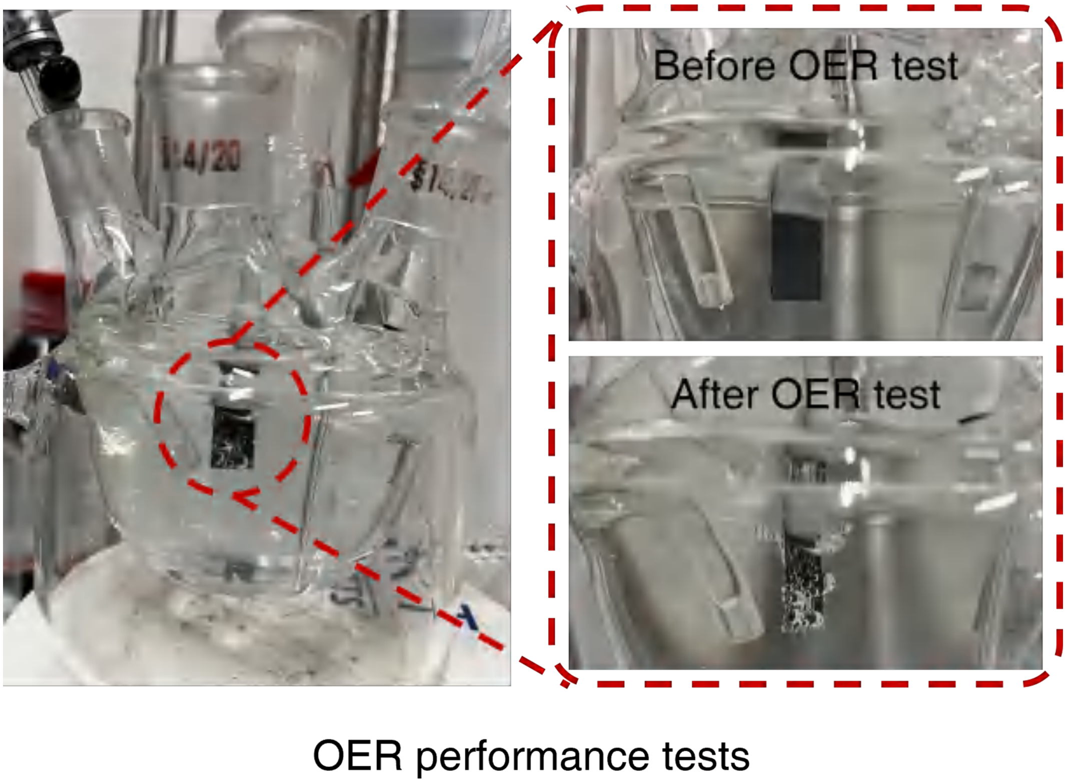


**Figure S24. OER testing process**. The three-electrode test was performed using a Chenhua electrochemical workstation and an electrode clamp. The catalyst was uniformly coated on a carbon paper, and the loading amount was 1 mg·cm$^{-2}$. After the LSV test, a large number of bubbles overflowed on the surface of the carbon paper, indicating that the material exhibited excellent OER catalytic performance.

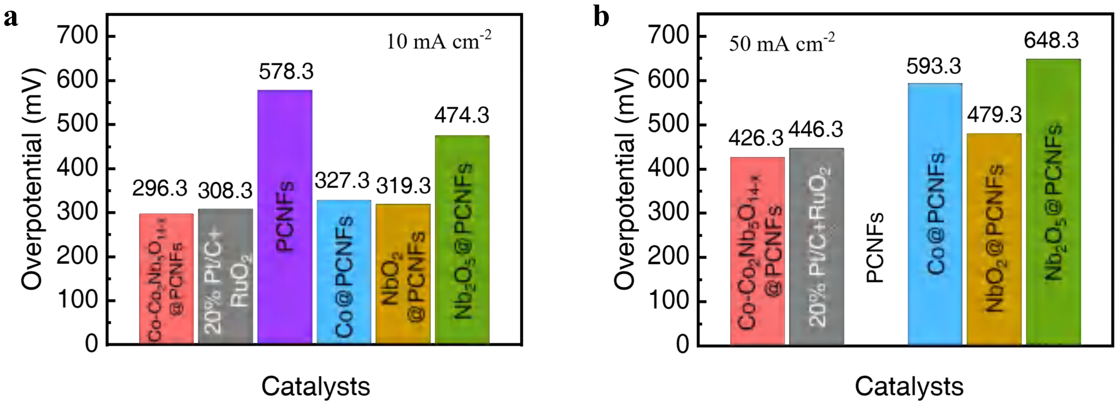


**Figure S25. Overpotentials of the six catalysts for better comparison**. (**a**) Overpotentials at 10 mA·cm$^{-2}$ and (**b**) at 50 mA·cm$^{-2}$. The data were obtained from the LSV curve measured in $O_2$-saturated 0.1 mol·L$^{-1}$ KOH solution. Lower Overpotential indicate better OER catalytic performance.

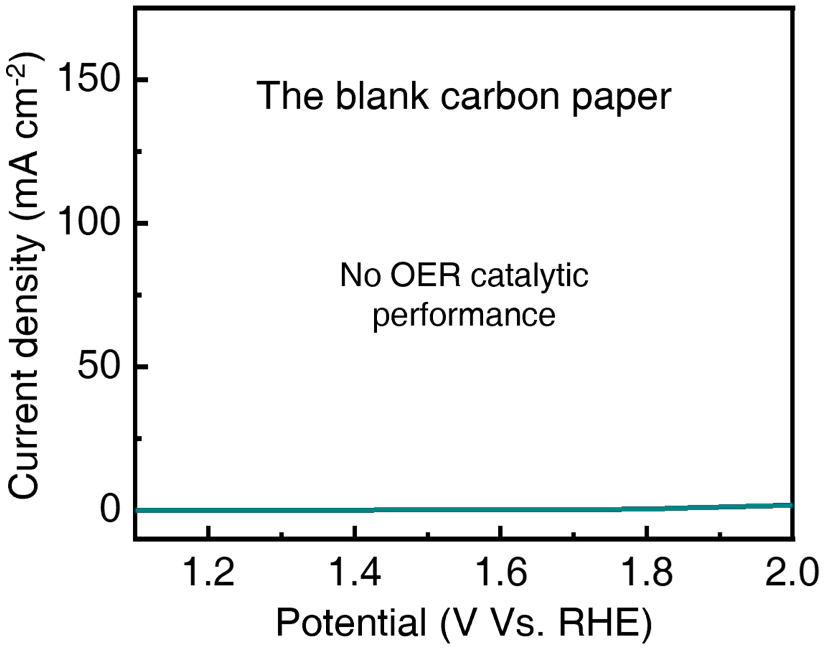


**Figure S26. LSV curve of the blank carbon paper**. It has almost no OER performance, so the influence of OER performance measured on this substrate could be excluded.

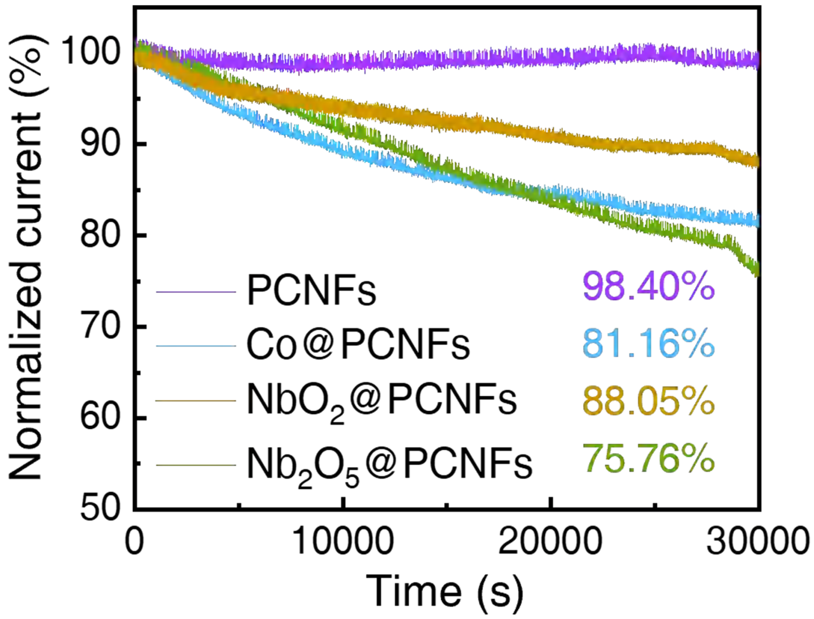


**Figure S27. i-t curves**. OER electrocatalytic stability testing of the catalysts at 1.6 V Vs. RHE. After 30000 s, the normalized current remained 98.4% (PCNFs), 81.16% (Co@PCNFs), 88.05% ($NbO_2$@PCNFs), and 75.76% ($Nb_2O_5$@PCNFs) of their initial currents, respectively.

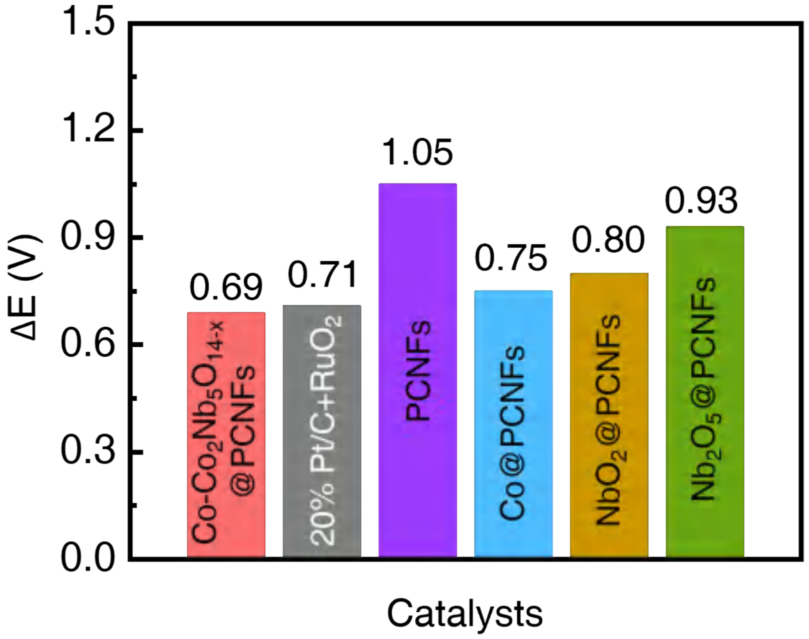


**Figure S28. Dual-functional indicator (ΔE) of the six catalysts for better comparison**. The data were obtained from the $E_{1/2}$ values measured at 1600 rpm and overpotentials at 10 mA·cm$^{-2}$. Lower ΔE values indicate better ORR/OER dual-functional catalytic performance.

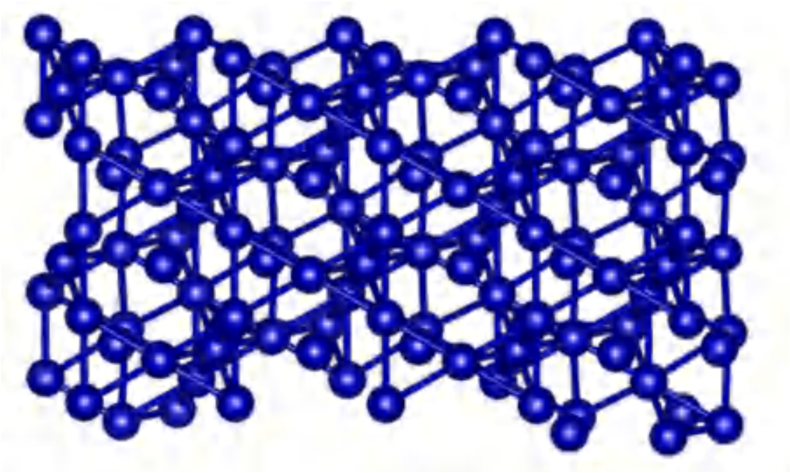

**Figure S29. DFT calculations.** Theoretical model of Co@PCNFs.

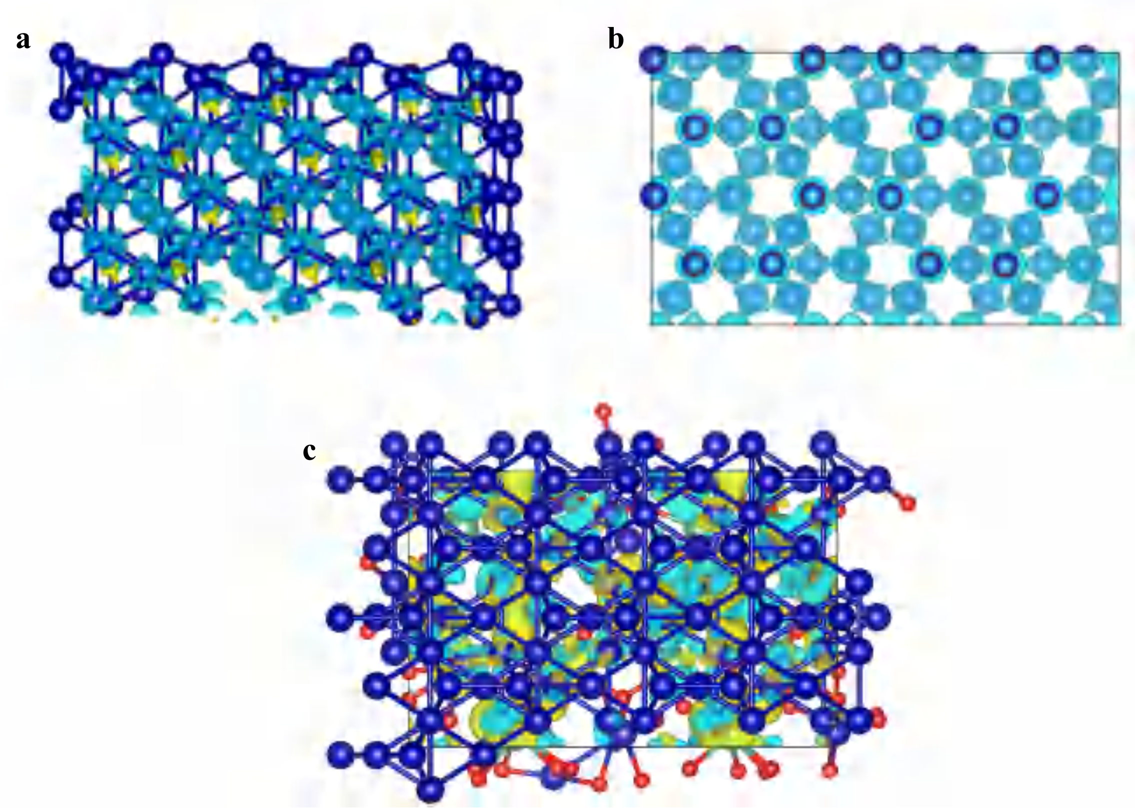


**Figure S30. DFT calculations**. (**a**) Side view and (**b**) top view of the charge density differences for Co@PCNFs. The following simulation calculations about Co@PCNFs were based on this model. (**c**) Top view of the charge density differences for Co-$Co_2Nb_5O_{14-x}$@PCNFs. A significant charge redistribution can be observed, indicating that it has better conductivity than Co@PCNFs. The following simulation calculations about Co-$Co_2Nb_5O_{14-x}$@PCNFs were based on this model.

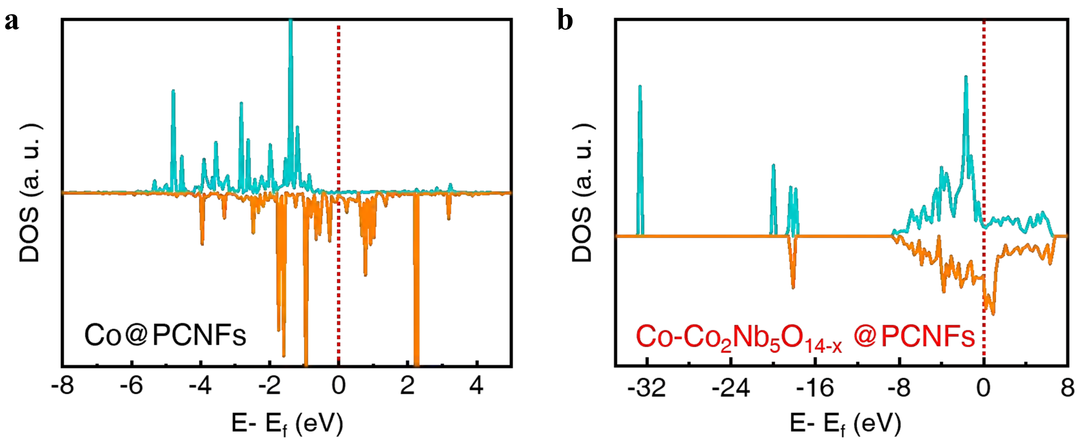


**Figure S31. DFT calculations**. DOS of the Co@PCNFs and Co-$Co_2Nb_5O_{14-x}$@PCNFs theoretical models. Both models show that the DOS crosses the Fermi level (E- Ef= 0), indicating overlapping conduction and valence bands, which is characteristic of typical metallic behavior.

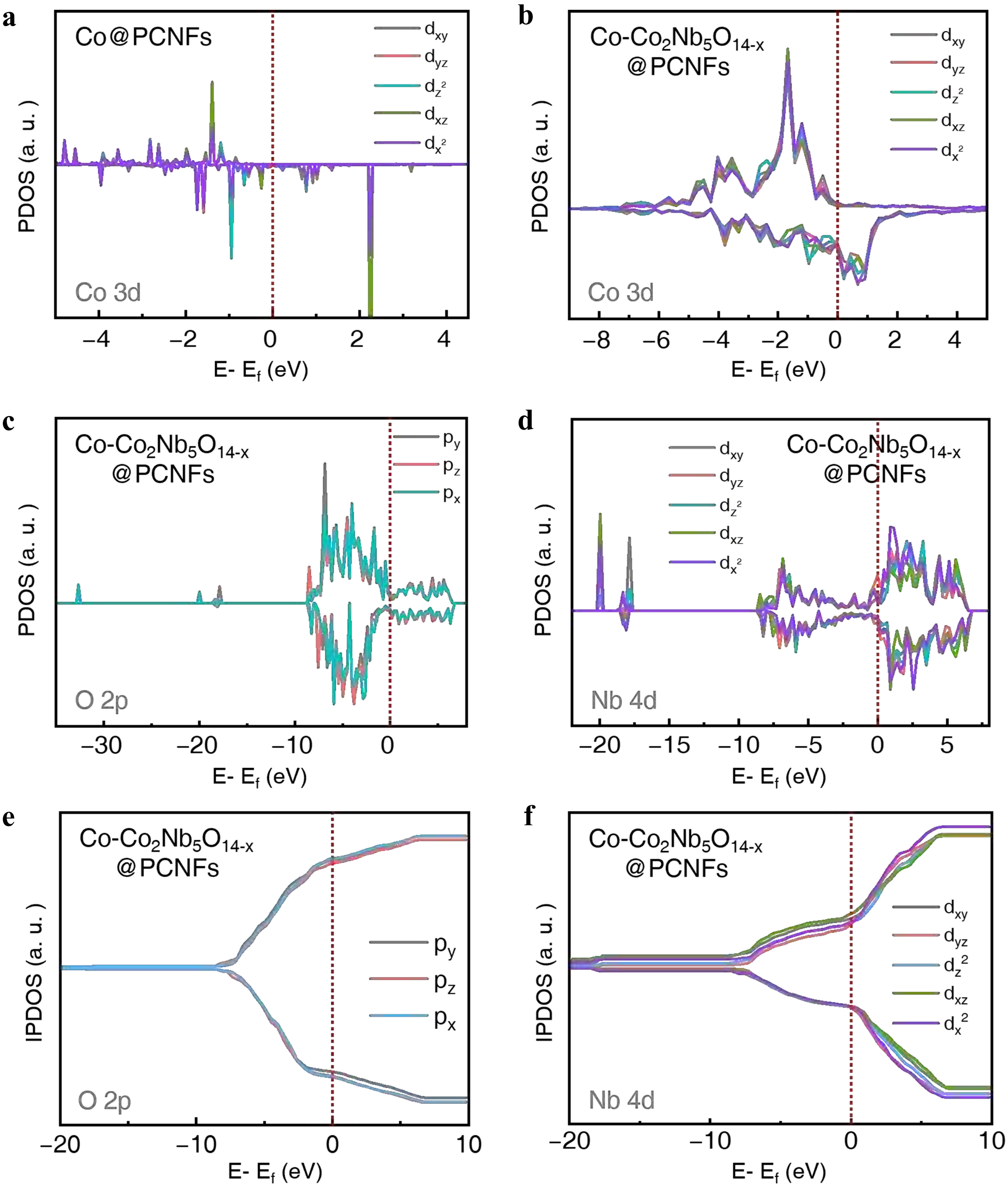


**Figure S32. DFT calculations**. DOS and IPDOS of the Co@PCNFs and Co-$Co_2Nb_5O_{14-x}$@PCNFs theoretical models. Compared to Co@PCNFs, the energy difference between the Co 3d orbitals in Co-$Co_2Nb_5O_{14-x}$@PCNFs decreased, and the energies of the d orbitals trended to a similar level.

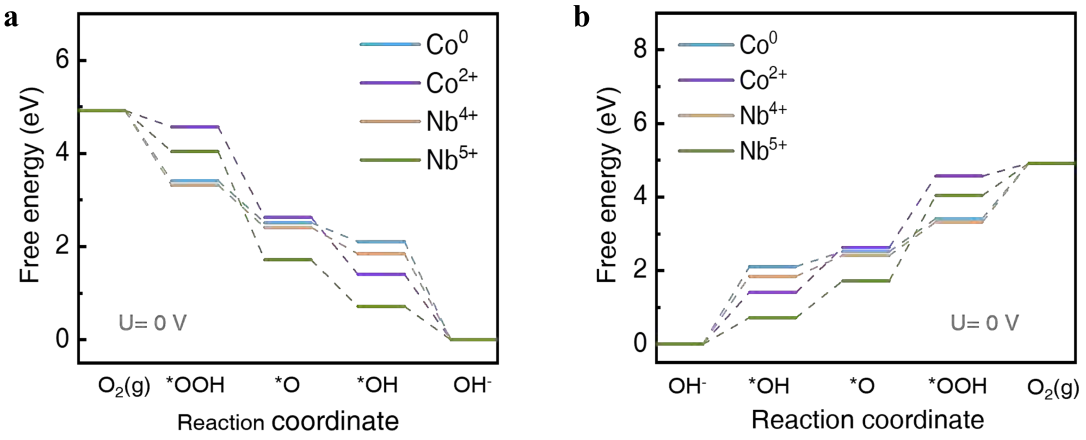


**Figure S33. DFT calculations.** Gibbs free energy diagrams for four sites of Co-$Co_2Nb_5O_{14-x}$@PCNFs in the (**a**) ORR and (**b**) OER process. The overpotential was set to U = 0 V. During the ORR process, the free energy of the four sites exhibited a downward trend, suggesting that the reaction proceeded spontaneously. Conversely, in the OER process, the free energy of the four sites exhibited an upward trend.

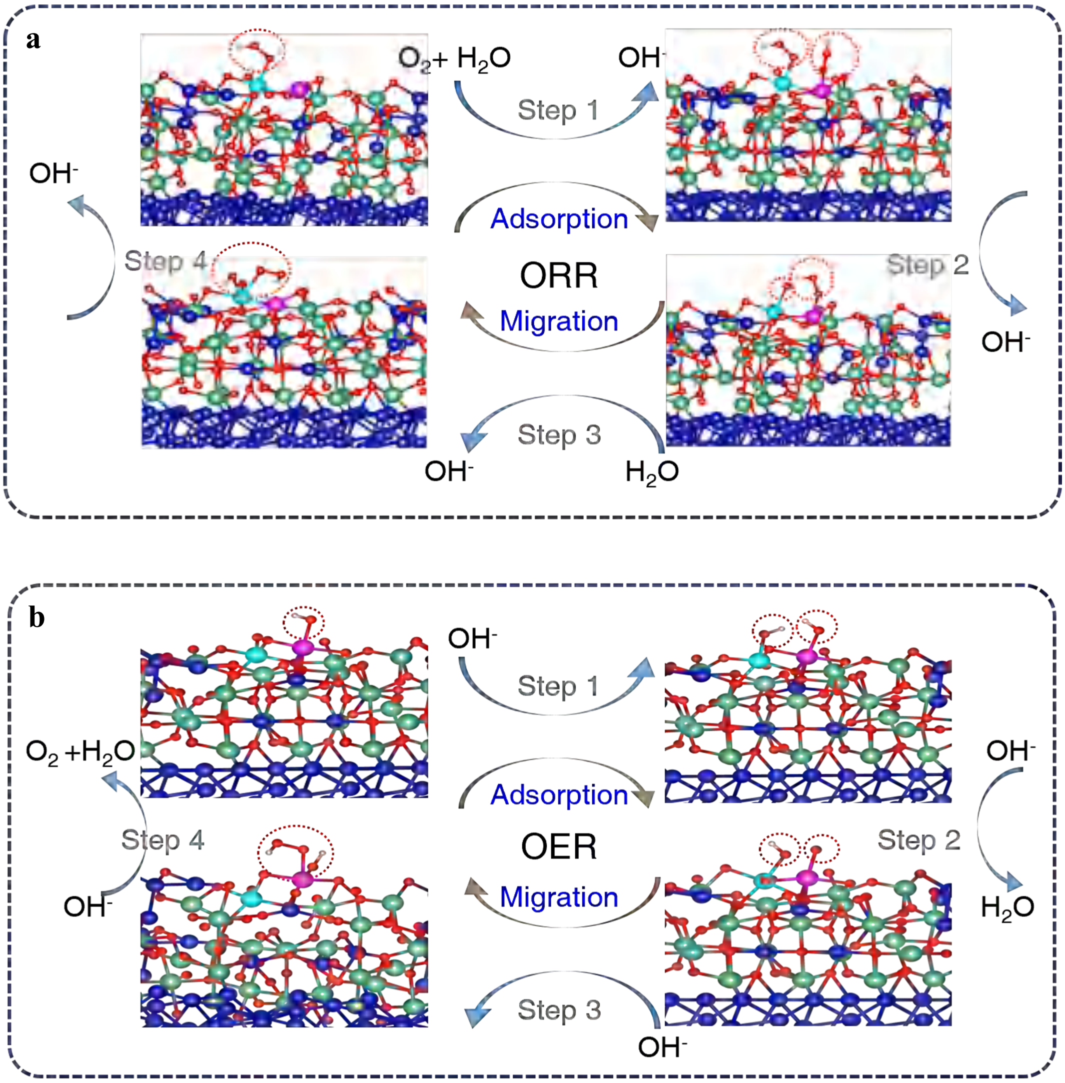


**Figure S34. Schematic diagram of the O species adsorption evolution mechanism**. The optimal adsorption and transfer paths of O intermediates in the ORR and OER processes were identified. The light blue atom represents $Nb^{5+}$, and the purple atom represents $Nb^{4+}$.

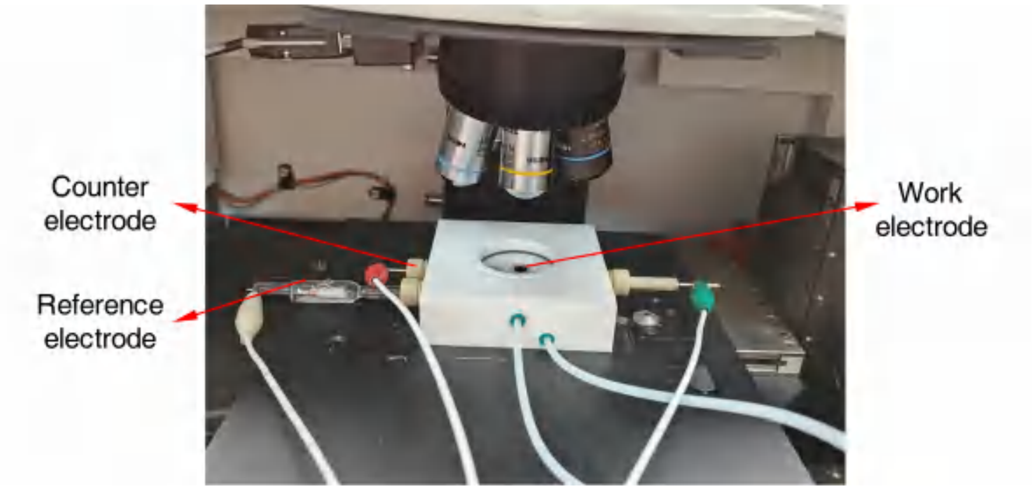


**Figure S35. Experimental setup of in-situ electrochemical Raman**. The test was conducted in 0.1 M KOH saturated with $O_2$.

a

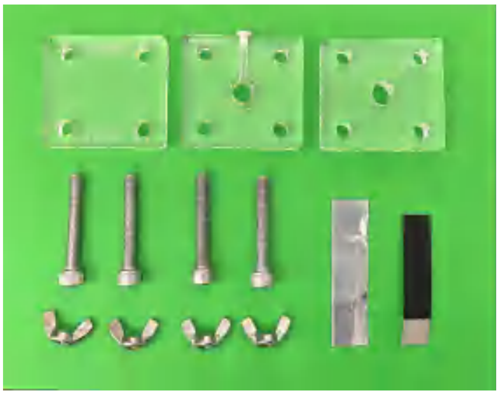

b

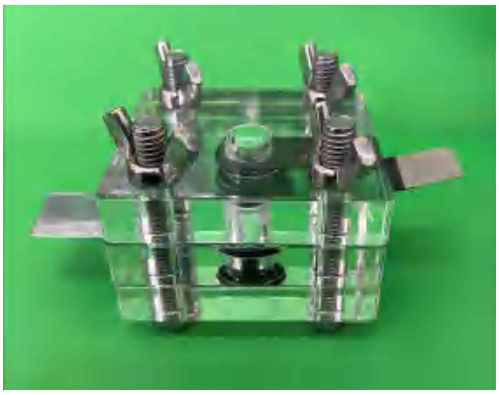

**Figure S36. Assembled the liquid ZAB**. The catalyst coated at a loading of 1 $mg\cdot cm^{-2}$ on a nickel foam and carbon paper composite cathode. A zinc sheet foil was used as the negative electrode, and the electrolyte consisted of 6 M KOH and 0.2 M $Zn(Ac)_2$.

a b

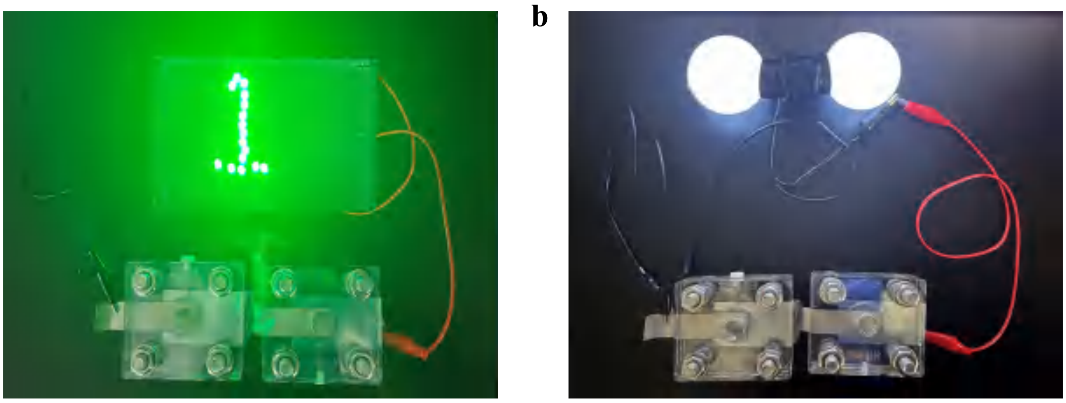

**Figure S37. Function display**. Two ZABs connected in series can successfully light up an LED cluster or two bulbs.

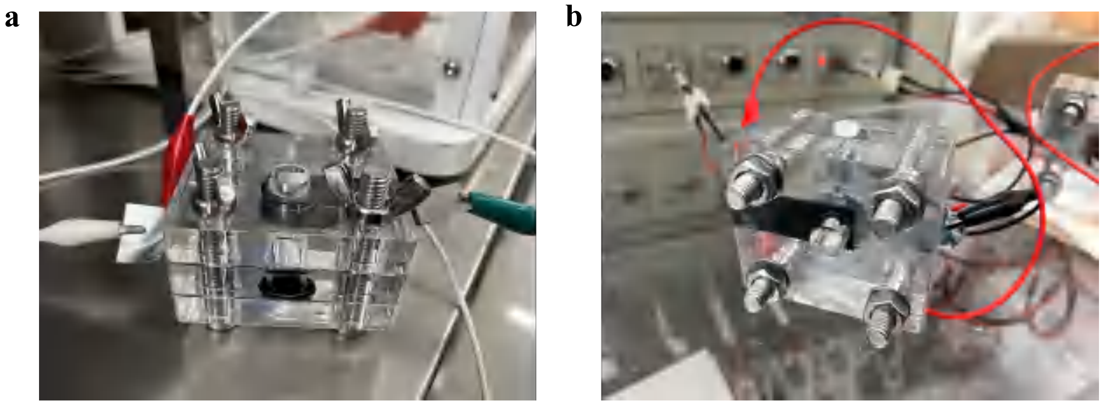


**Figure S38. Performance test method of ZABs.** (**a**) The three-electrode test system was used to measure the open-circuit voltage and polarization curves, with Chenhua electrochemical workstation as the testing equipment. (**b**) The Land test system was employed to measure the specific capacity and cycle stability of the ZAB.

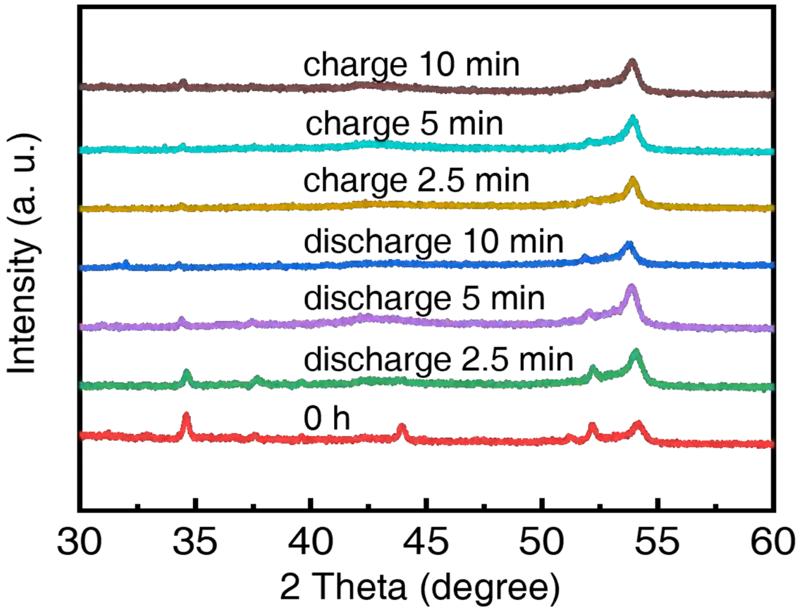


**Figure S39. XRD analysis of cathode materials during charging and discharging**. During the discharge process, the peak intensities of $Co_2Nb_5O_{14-x}$ (1 0 1) at 34.6° and Co (200) at 51.9° gradually decreased. These peak intensities then increased again during the charging process. This phenomenon confirmed the reversible dynamic changes in the Co-$Co_2Nb_5O_{14-x}$@PCNFs catalyst material during the charging and discharging processes of the ZAB, and explains the reason of the superlong stable cycle of the ZAB.

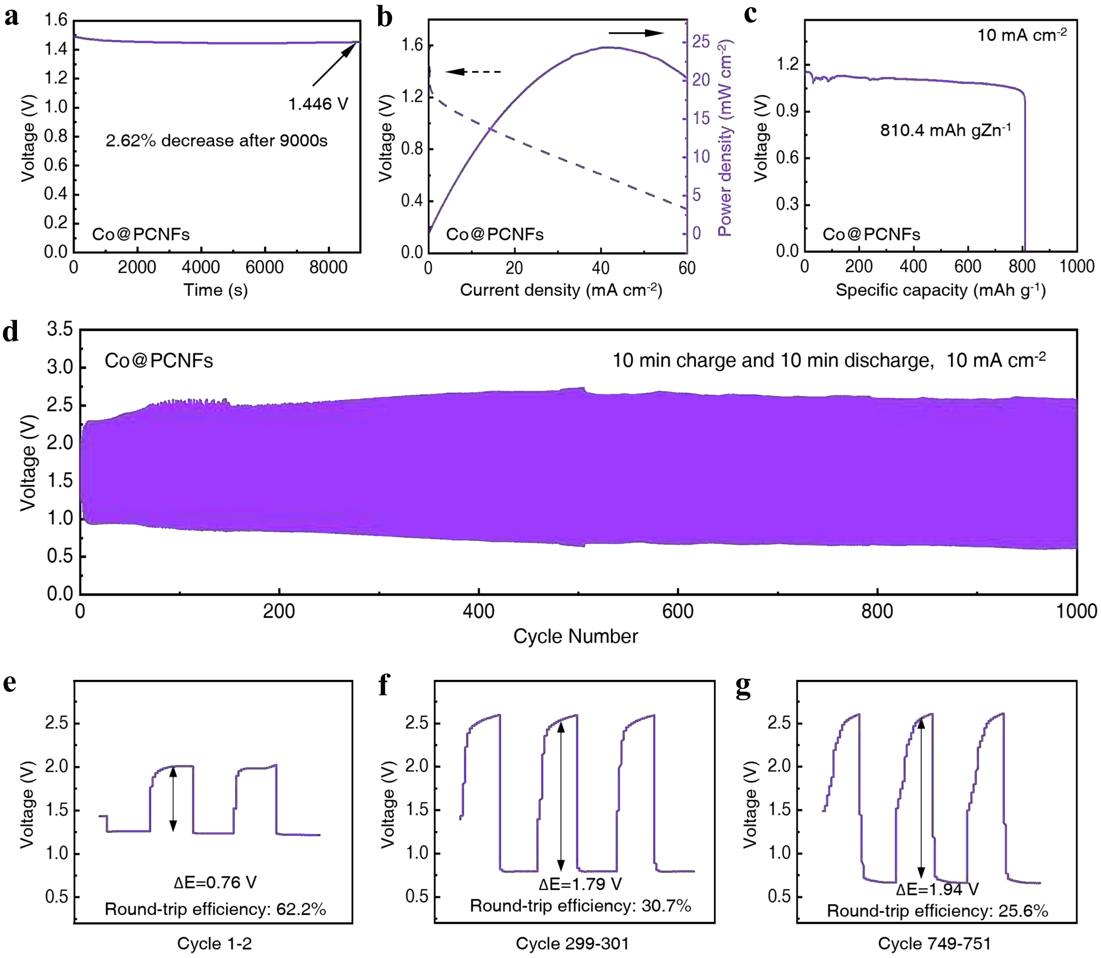


**Figure S40. Electrochemical performance of Co@PCNFs liquid ZAB**. (**a**) Open-circuit voltage plots of initial ZAB assembled with Co@PCNFs. (**b**) Discharge polarization curves and power densities, and (**c**) discharge curves at 10 mA·cm$^{-2}$ of rechargeable liquid ZAB with of the Co@PCNFs catalysts. (**d**) Long-term cycling performance of Co@PCNFs-based ZAB at 10 mA·cm$^{-2}$. The ZAB performances at (**e**) cycle 1, (**f**) cycle 300, and (**g**) cycle 750.

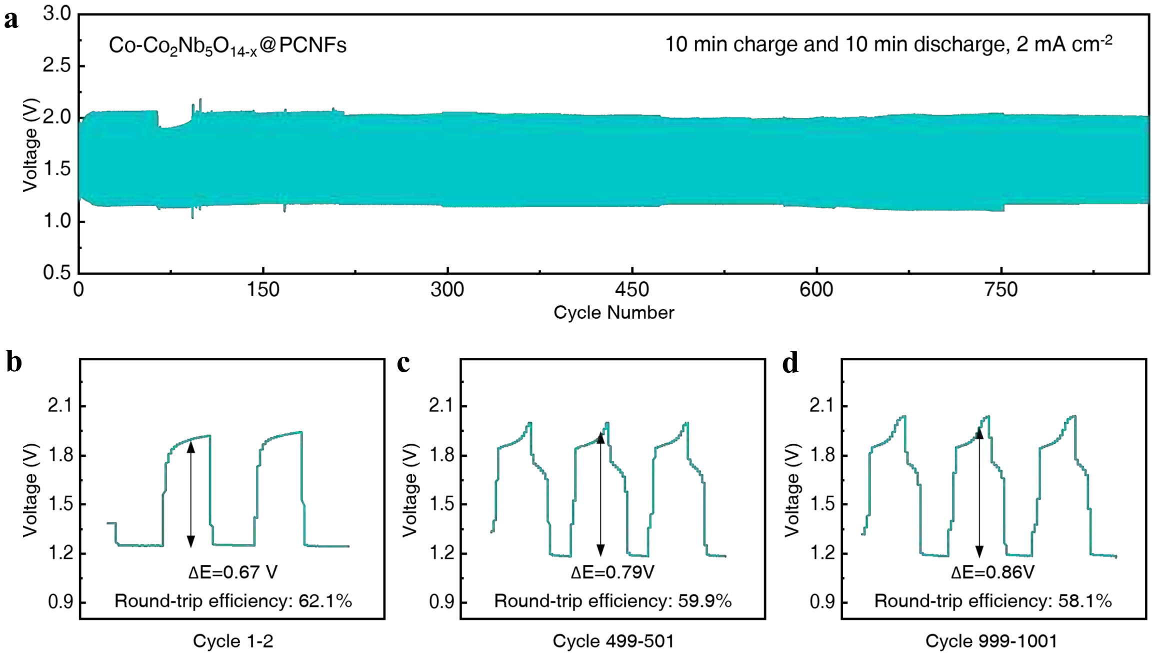


**Figure S41. Long-term cycling performance of Co-$Co_2Nb_5O_{14-x}$@PCNFs-based ZAB at 2 mA·cm$^{-2}$.** After 900 cycles, it still maintained a high round-trip efficiency of 58.1% and a low voltage gap of 0.86 V. The voltage dip around 60 cycles was due to the leak of ZAB electrolyte. Overall, the cycling performance of Co-$Co_2Nb_5O_{14-x}$@PCNFs-based ZAB at 2 mA·cm$^{-2}$ was comparable to that of 20% Pt/C+ $RuO_2$ and was superior to that of Co@PCNFs.

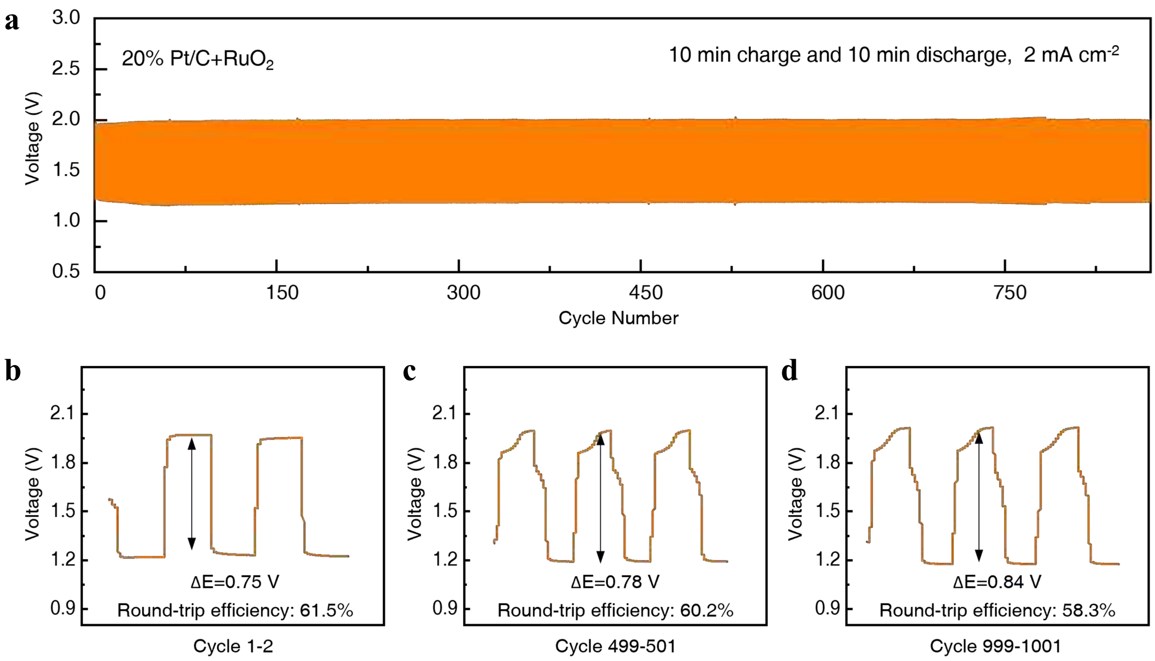


**Figure S42. Long-term cycling performance of 20% Pt/C+ $RuO_2$-based ZAB at 2 mA·cm$^{-2}$.** After 900 cycles, it maintained a round-trip efficiency of 58.3% and a voltage gap of 0.84 V. The cycling performance was comparable to that of Co-$Co_2Nb_5O_{14-x}$@PCNFs-based ZAB.

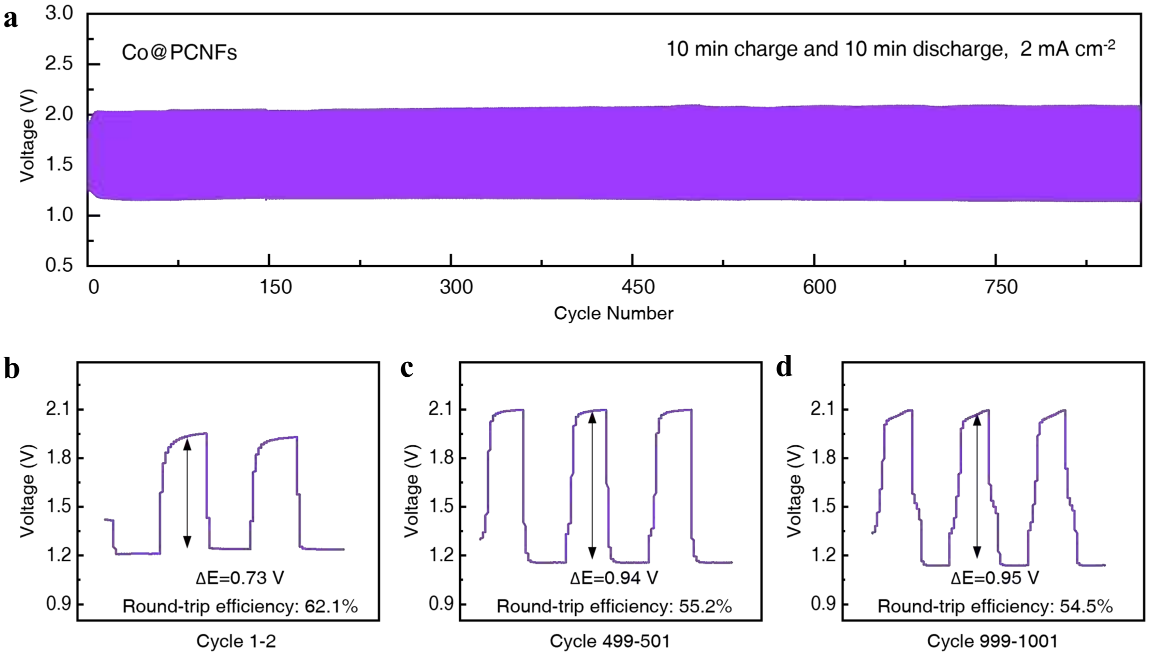


**Figure S43. Long-term cycling performance of Co@PCNFs-based ZAB at 2 mA·cm$^{-2}$**. After 900 cycles, it had a round-trip efficiency of 54.5% and a voltage gap of 0.95 V. The cycling performance was inferior to that of Co-$Co_2Nb_5O_{14-x}$@PCNFs-based ZAB.

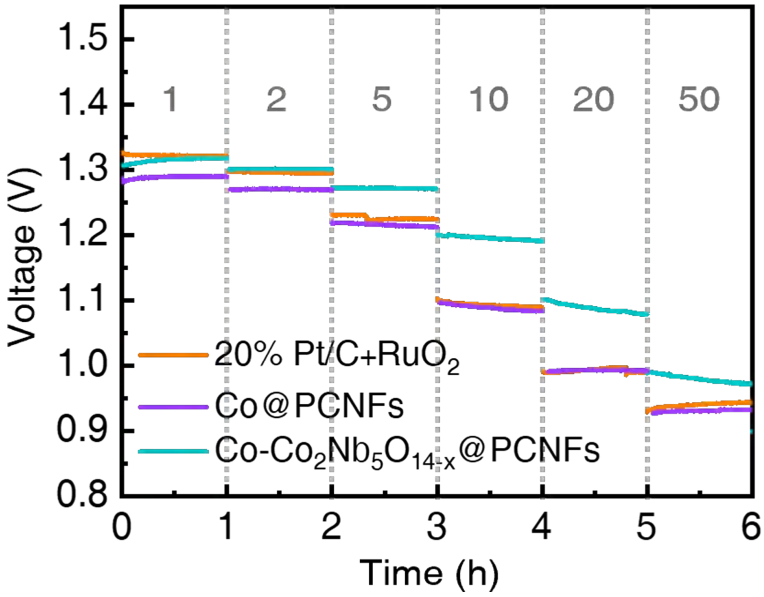


**Figure S44. Discharge curves at various current densities (mA·cm$^{-2}$)**. The discharge potential plateaus of the ZABs based on Co-$Co_2Nb_5O_{14-x}$@PCNFs and 20% Pt/C+ $RuO_2$ were not much different at lower current densities, but they were more stable and higher for Co-$Co_2Nb_5O_{14-x}$@PCNFs than that for 20% Pt/C+ $RuO_2$ at higher current densities. And the discharge potential plateaus for Co@PCNFs maintained the lowest of the three**.**

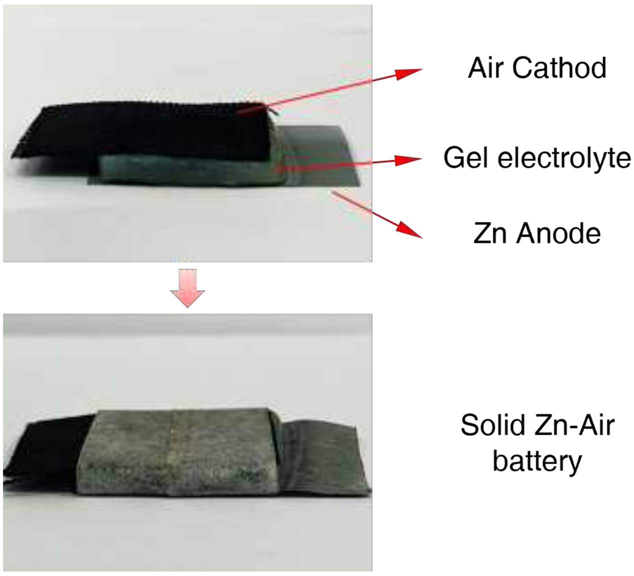


**Figure S45. Integration of solid ZAB**. It was using carbon cloth loaded with Co-$Co_2Nb_5O_{14-x}$@PCNFs as the air cathode, alkaline polyvinyl alcohol (PVA) gel as the electrolyte, and Zn foil as the anode. Waterproof and breathable tapes were used to encapsulate the ZAB to ensure the proper entry and exit of oxygen.

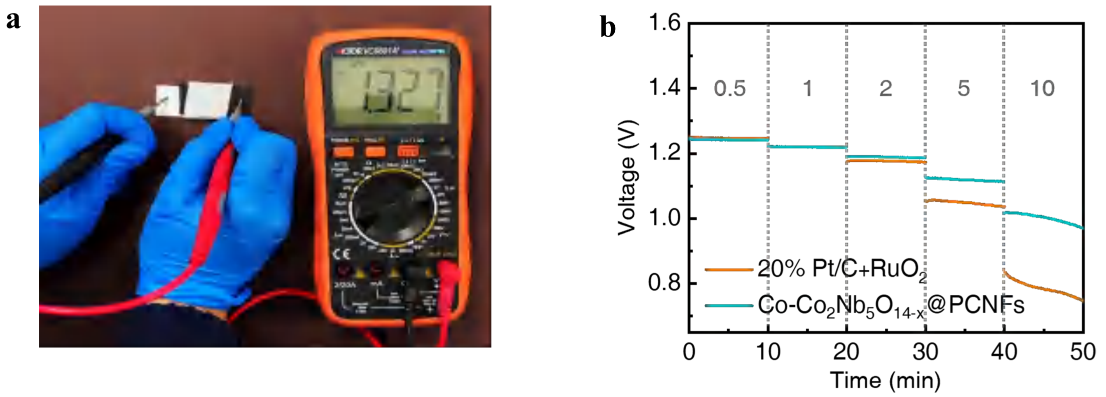


**Figure S46.** (**a**) OCV of 20% Pt/C+ $RuO_2$-based solid ZAB and (**b**) Discharge curves at various current densities (mA·cm$^{-2}$). The discharge voltage of Co-$Co_2Nb_5O_{14-x}$@PCNFs gradually decreased from 1.24 to 1.00 V (the average value of each segment) with the current density varying from 0.5 to 10 mA·cm$^{-2}$, which was superior to that of commercial catalysts (1.24 to 0.78 V).

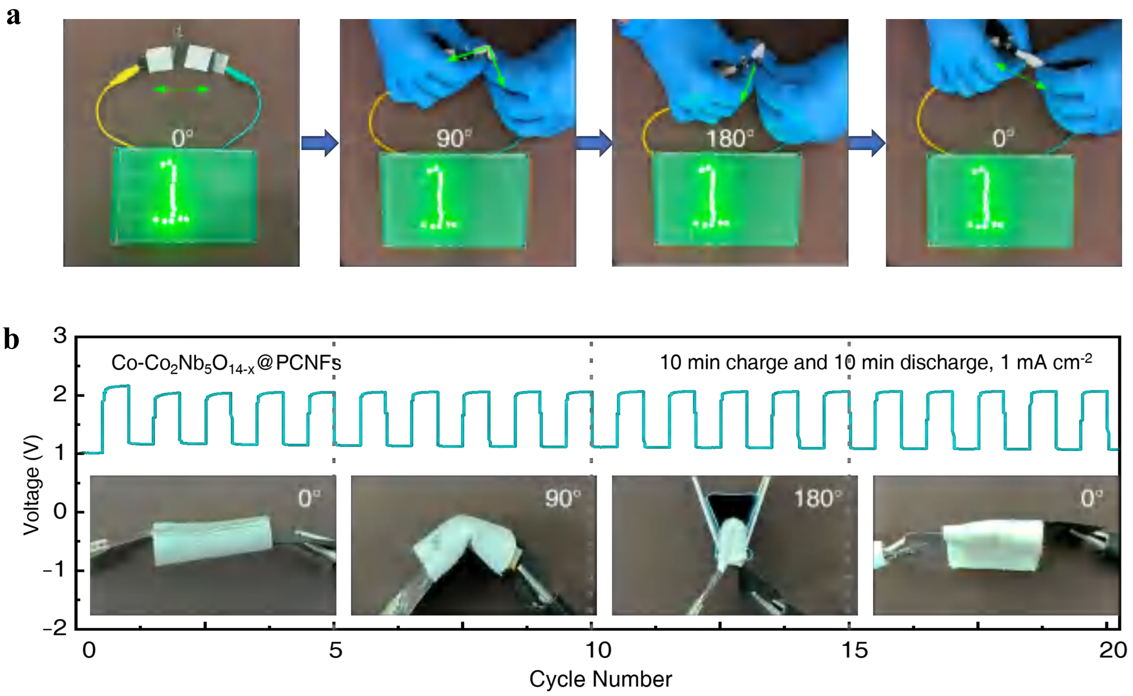


**Figure S47. Flexibility performance of solid ZAB.** (**a**) The LED displays. (**b**)Charge and discharge curve of solid ZAB at 1 mA·cm$^{-2}$. Bending the ZAB at different angles (0°, 90°, 180°, unfolded) showed no noticeable changes in the charge and discharge, and it could effortlessly illuminate the LED display.

## Supporting Tables

**Table S1. Detailed results of ICP for Co-$Co_2Nb_5O_{14-x}$@PCNFs.**

| No. | Sample Quality $m_0$ (g) | Constant Volume $V_0$ (mL) | Element | Element Concentration in Solution $C_0$ (mg/L) | Dilution Factor f | Initial Solution Element Concentration of the Digestion Solution $C_1$ (mg/L) | Element Content $C_x$ (mg/kg) | Element Weight Content W (%) |
|---|---|---|---|---|---|---|---|---|
| 1 | 0.0482 | 25 | Co | 2.309 | 100 | 230.9316525 | 119777.8 | 11.98% |
| 2 | 0.0482 | 25 | Co | 2.309 | 100 | 230.8842079 | 119753.2 | 11.98% |
| 3 | 0.0482 | 25 | Nb | 2.566 | 100 | 256.590725 | 133086.5 | 13.31% |
| 4 | 0.0482 | 25 | Nb | 2.568 | 100 | 256.760231 | 133174.4 | 13.32% |

**Table S2. Comparison of bifunctional catalytic performance with other reported Co-based catalysts**

| No. | Catalyst | $E_{j=10}$/ V | $E_{1/2}$/V | ΔE | Ref. |
|---|---|---|---|---|---|
| 1 | Co-$Co_2Nb_5O_{14-x}$ @PCNFs | 1.5263 | 0.84 | 0.69 | This Work |
| 2 | 20% Pt/C+ $RuO_2$ | 1.5383 | 0.83 | 0.71 | This Work |
| 3 | Co@C-O-Cs | 1.524 | 0.82 | 0.704 | 7 |
| 4 | H-NSC@ Co/NSC | 1.60 | 0.85 | 0.75 | 8 |
| 5 | Co/SP-NC | 1.573 | 0.8603 | 0.7127 | 9 |
| 6 | Fe–Co(DSA) @3DNC | 1.65 | 0.816 | 0.834 | 10 |
| 7 | Co/MnO @N-DSC | 1.54 | 0.84 | 0.70 | 11 |
| 8 | CoS/CoO@ NGNs | 1.61 | 0.84 | 0.77 | 12 |
| 9 | CoNi/NHCS-TUC-3 | 1.638 | 0.832 | 0.806 | 13 |
| 10 | CoTAPP-PATA-COF | 1.65 | 0.801 | 0.849 | 14 |
| 11 | Co-$CeO_2$/C | 1.61 | 0.75 | 0.86 | 15 |
| 12 | $Co_2P$/Co-NC | 1.599 | 0.88 | 0.719 | 16 |
| 13 | CoNC-CNF-1000 | 1.68 | 0.8 | 0.88 | 17 |
| 14 | Co/CoO@NSC | 1.61 | 0.835 | 0.775 | 18 |
| 15 | Mo─Co/N─C | 1.579 | 0.869 | 0.71 | 19 |

**Table S3. Performance comparison of Co-based liquid ZAB.**

| No. | Catalyst | OCV (V) | Specific capacity (mAh $g_{Zn}^{-1}$ ) | Power density (mW $cm^{-2}$) | Cycle number | Ref. |
|---|---|---|---|---|---|---|
| 1 | Co-$Co_2Nb_5O_{14-x}$ @PCNFs | 1.496 | 850.6 | 114.07 | 2050 | This Work |
| 2 | 20% Pt/C+ $RuO_2$ | 1.458 | 769.9 | 66.08 | 270 | This Work |
| 3 | Co@C–O–Cs | 1.426 | 720.7 | 106.4 | 750 | 7 |
| 4 | CoN/$Co_3O_4$ HNPs@ NCNWs | 1.46 | 779.8 | 118.3 | 200 | 20 |
| 5 | $Fe_1Co_3$–NC-1100 | 1.479 | 700 | 372 | 570 | 21 |
| 6 | $CoSe_2$/CoNC | 1.54 | 835 | 165 | 400 | 22 |
| 7 | Co/SP-NC | 1.46 | 801 | 187 | 840 | 9 |
| 8 | Co/MnO@ N-DSC | 1.52 | 986.1 | 172.7 | 420 | 11 |
| 9 | CoS/CoO@ NGNs | 1.45 | 723.9 | 137.8 | 300 | 12 |
| 10 | CoNi/NHCS-TUC-3 | 1.596 | - | 255.9 | 142 | 13 |
| 11 | $Co_2P$/Co-NC | 1.45 | 837 | 187 | 420 | 16 |
| 12 | Co/CoO@NSC | 1.53 | 759.7 | 187.6 | 1200 | 18 |
| 13 | Fe-$Co_2P$ @NPDC | 1.438 | 762 | 340 | 498 | 23 |
| 14 | Co-$CoN_4$@ NCNs | 1.47 | 776.7 | 118.8 | 1500 | 24 |
| 15 | CoNP@ FeNC-0.05 | 1.51 | - | 104.4 | 500 | 25 |

**Table S4. Performance comparison of Co-based flexible solid ZAB.**

| No. | Catalyst | Electrolyte | OCV (V) | Power density (mW cm$^{-2}$) | Current density (mA cm$^{-2}$) | Cycling duration (min) | Cycle number | Ref. |
|---|---|---|---|---|---|---|---|---|
| 1 | $Co\text{-}Co_2Nb_5O_{14\text{-}x}$ @PCNFs | PVA-KOH | 1.398 | 68.4 | 2 | 20 | 160 | This Work |
| 2 | 20% Pt/C+ $RuO_2$ | PVA-KOH | 1.327 | 31.3 | 2 | 20 | 19 | This Work |
| 3 | Ultrathin $Co_3O_4$ | PVA–KOH | 1.52 | 108 | 2 | 20 | 30 | 26 |
| 4 | Co SA/NCFs | PVA-KOH | 1.42 | - | 2 | 20 | 90 | 27 |
| 5 | Co@C–O–Cs | PVA-KOH | 1.43 | 59.1 | 1 | 20 | 20 | 7 |
| 6 | CoNi-CoN4-HPC-900 | PVA-KOH | 1.50 | 116 | 2 | 10 | 90 | 28 |
| 7 | CC-A-N@Co-NCNT | PVA-KOH | 1.42 | 44 | 2 | 20 | 90 | 29 |
| 8 | $Co_{SAs\text{-}NPs}$/NC | PAA-KOH | 1.492 | 73.4 | 1 | 20 | 110 | 30 |
| 9 | CoN/ $Co_3O_4$ HNPs@NCNWs | PAA-KOH-ZnO | 1.34 | 62 | 1 | 20 | 24 | 20 |
| 10 | $CuCo_2S_4$NSs | PVA-KOH | 1.20 | - | 1 | 20 | 66 | 31 |
| 11 | Co-Nx-YSC-600/CC | Alkaline PVA | 1.345 | 55.3 | 5 | 20 | 12 | 32 |
| 12 | $Fe_1Co_3$–NC-1100 | PAA-KOH | - | - | 2 | 20 | 16 | 21 |
| 13 | $CoSe_2$/CoNC | PAA-CMC-KOH | 1.51 | 112 | 2 | 10 | 120 | 22 |
| 14 | Co-N-CCNFMs/CC | PANa-KOH | 1.461 | 61.5 | 2 | 20 | 108 | 33 |

**Table S5. Benchmarking against the liquid ZABs based on Pt/C+ $RuO_2$.**

| No. | Catalyst | OCV (V) | Specific capacity (mAh $g_{Zn}^{-1}$) | Power density (mW $cm^{-2}$) | Cycle number | Ref. |
|---|---|---|---|---|---|---|
| 1 | Co-$Co_2Nb_5O_{14-x}$ @PCNFs | 1.496 | 850.6 | 114.07 | 2050 | This Work |
| 2 | 20% Pt/C+ $RuO_2$ | 1.458 | 769.9 | 66.08 | 270 | This Work |
| 3 | 20% Pt/C+ $RuO_2$ | 1.40 | 690.7 | 49.4 | 300 | 7 |
| 4 | 20% Pt/C+ $RuO_2$ | 1.43 | 695.5 | 102.2 | 200 | 20 |
| 5 | Pt/C+ $RuO_2$ | 1.51 | 751.4 | 120 | 150 | 22 |
| 6 | 20% Pt/C+ $RuO_2$ | 1.42 | 779.4 | - | ~360 | 9 |
| 7 | 20%Pt/C+ $RuO_2$ | 1.40 | 816 | 180 | - | 16 |
| 8 | 20%Pt/C+ $RuO_2$ | - | 714.3 | 161 | 300 | 18 |
| 9 | Pt/C+ $RuO_2$ | - | 651 | 165 | - | 23 |
| 10 | 20% Pt/C+ $RuO_2$ | 1.40 | 691.2 | 91.4 | 300 | 24 |
| 11 | Pt/C+ $RuO_2$ | 1.39 | - | 60.4 | 237 | 25 |
| 12 | 20% Pt/C+ $RuO_2$ | - | 729.8 | 178 | 180 | 34 |
| 13 | 20% Pt/C+ $RuO_2$ | 1.44 | 769.5 | 118.5 | - | 35 |
| 14 | 20% Pt/C+ $RuO_2$ | - | 756 | 118.97 | 150 | 36 |
| 15 | 20% Pt/C+ $RuO_2$ | 1.45 | 762 | 127 | - | 37 |

## References

1. G. Kresse, D. Joubert, *Phys. Rev. B* **1999**, *59*, 1758.

2. J. Perdew, K. Burke, M. Ernzerhof, *Phys. Rev. Lett.* **1996**, *77*, 3865.

3. V. Wang, N. Xu, J. Liu, G. Tang, W. Geng, *Comput. Phys. Commun.* **2021**, *267*, 108033.

4. W. Tang, E. Sanville, G. Henkelman, *J. Phys-Condens. Mat.* **2009**, *21*, 0842.

5. K. Momma, F. Izumi, *J. Appl. Crystallogr.* **2008**, *41*, 653.

6. S. Zhao, K. Wang, X. Zou, L. Gan, H. Du, C. Xu, F. Kang, W. Duan, J. Li, *Nano Res.* **2019**, *12*, 925.

7. K. Ding, Y. Ye, J. Hu, L. Zhao, W. Jin, J. Luo, S. Cai, B. Weng, G. Zou, H. Hou, X. Ji, *Nano-Micro Lett.* **2023**, *15*, 28.

8. W. Li, J. Wang, J. Chen, K. Chen, Z. Wen, A. Huang, *Small* **2022**, *18*, 2202018.

9. H. Chang, X. Liu, S. Zhao, Z. Liu, R. Lv, Q. Zhang, T. Yi, *Adv. Func. Mater.* **2024**, *34*, 2313491.

10. X. Li, H. Liu, Y. Sun, L. Zhu, X. Yin, S. Sun, Z. Fu, Y. Lu, X. Wang, Z. Cheng, *Adv. Sci.* **2020**, *7*, 2002242.

11. X. Guo, Y. Yuan, S. Li, J. Wang, Z. Bai, L. Yang, *Int. J. Hydrogen Energy* **2023**, *48*, 26805.

12. Y. Tian, L. Xu, M. Li, D. Yuan, X. Liu, J. Qian, Y. Dou, J. Qiu, S. Zhang, *Nano-Micro Lett.* **2020**, *13*, 3.

13. K. Sheng, Q. Yi, A. Chen, Y. Wang, Y. Yan, H. Nie, X. Zhou, *ACS Appl. Mater. Interfaces* **2021**, *13*, 45394.

14. M. Liu, S. Liu, C. Cui, Q. Miao, Y. He, X. Li, Q. Xu, G. Zeng, *Angew. Chem. Int. Ed.* **2022**, *134*, e202213522.

15. Z. Liu, J. Wan, M. Li, Z. Shi, J. Liu, Y. Tang, *Nanoscale* **2022**, *14*, 1997.

16. X. Liu, J. Wu, Z. Luo, P. Liu, Y. Tian, X. Wang, H. Li, *ACS Appl. Mater. Interfaces* **2023**, *15*, 9240.

17. W. Zhang, X. Yao, S. Zhou, X. Li, L. Li, Z. Yu, L. Gu, *Small* **2018**, *14*, 1800423.

18. D. Zhou, H. Fu, J. Long, K. Shen, X. Gou, *J. Energy Chem.* **2022**, *64*, 385.

19. Y. Zhang, X. Ma, Z. Cheng, J. Wang, G. Wen, L. Yang, Z. Bai, *Adv. Func. Mater.* **2024**, *34*, 2314622.

20. Q. Zhou, S. Zhang, G. Zhou, H. Pang, M. Zhang, L. Xu, K. Sun, Y. Tang, K. Huang, *Small* **2023**, *19*, 2301324.

21. Y. He, X. Yang, Y. Li, L. Liu, S. Guo, C. Shu, F. Liu, Y. Liu, Q. Tan, G. Wu, *ACS Catal.* **2022**, *12*, 1216.

22. Q. Xu, L. Peng, K. Luo, J. Zhong, C. Zhang, D. Yuan, *J. Colloid Interf. Sci.* **2023**, *643*, 73.

23. L. Li, L. Zhang, Z. Nie, W. Ma, N. Li, T. Wågberg, G. Hu, *J. Mater. Chem. A* **2022**, *10*, 21659.

24. K. Ding, J. Hu, J. Luo, L. Zhao, W. Jin, Y. Liu, Z. Wu, G. Zou, H. Hou, X. Ji, *Adv. Func. Mater.* **2022**, *32*, 2207331.

25. Y. Xue, Y. Guo, Q. Zhang, Z. Xie, J. Wei, Z. Zhou, *Nano-Micro Lett.* **2022**, *14*, 162.

26. X. Chen, B. Liu, C. Zhong, Z. Liu, J. Liu, L. Ma, Y. Deng, X. Han, T. Wu, W. Hu, J. Lu, *Adv. Energy Mater.* **2017**, *7*, 1700779.

27. Y. Han, H. Duan, C. Zhou, H. Meng, Q. Jiang, B. Wang, W. Yan, R. Zhang, *Nano Lett.* **2022**, *22*, 2497.

28. Y. Liu, Z. Chen, Z. Li, N. Zhao, Y. Xie, Y. Du, J. Xuan, D. Xiong, J. Zhou, L. Cai, Y. Yang, *Nano Energy* **2022**, *99*, 107325.

29. Q. Lu, X. Zou, K. Liao, R. Ran, W. Zhou, M. Ni, Z. Shao, *Carbon Energy* **2020**, *2*, 461.

30. Y. Yang, Y. Xiao, L. Zhang, H. Wang, K. Chen, W. Lin, N. Jin, C. Sun, Y. Shao, J. Chen, J. Qian, L. Han, *Appl. Catal. B: Environ.* **2024**, *347*, 123792.

31. Y. Li, J. Yin, L. An, M. Lu, K. Sun, Y. Zhao, F. Cheng, P. Xi, *Nanoscale* **2018**, *10*, 6581.

32. Z. Li, J. Yang, X. Ge, Y. Deng, G. Jiang, H. Li, G. Sun, W. Liu, Y. Zheng, H. Dou, H. Jiao, J. Zhu, N. Li, Y. Hu, M. Feng, Z. Chen, *Nano Energy* **2021**, *89*, 106314.

33. Z. Xu, J. Zhu, J. Shao, Y. Xia, J. Tseng, C. Jiao, G. Ren, P. Liu, G. Li, R. Chen, S. Chen, F. Huang, H. Wang, *Energy Storage Mater.* **2022**, *47*, 365.

34. Z. Zheng, C. Wang, P. Mao, Y. Zhu, R. Ran, W. Zhou, K. Liao, Z. Shao, *Carbon Energy* **2023**, *5*, e274.

35. H. Zhang, H. Chen, S. Feizpoor, L. Li, X. Zhang, X. Xu, Z. Zhuang, Z. Li, W. Hu, R. Snyders, D. Wang, C. Wang, *Adv. Mater.* **2024**, *36*, 2400523.

36. J. Chen, C. Qiu, L. Zhang, B. Wang, P. Zhao, Y. Zhao, H. Wang, G. Yang, A. Sun, J. Fan, Q. Xv, O. J. Rojas, *Energy & Environ. Sci.* **2024**, *17*, 4746.

37. W. Zhang, C. Xu, H. Zheng, R. Li, K. Zhou, *Adv. Func. Mater.* **2022**, *32*, 2200763.
